# Impact of Pulsed CO_2_ Electrolysis on Surface Structures and Product Distribution for Oxide-Derived Cu

**DOI:** 10.1021/acs.jpcc.6c05013

**Published:** 2026-10-19

**Authors:** Bianca Ligt, Jérôme F. M. Simons, Jason M. J. J. Heinrichs, Laura B. Donk, Marta Costa Figueiredo, Emiel J. M. Hensen

**Affiliations:** † Laboratory of Inorganic Materials and Catalysis, Department of Chemical Engineering and Chemistry, 3169 Eindhoven University of Technology, P.O. Box 513, 5600 MB Eindhoven, The Netherlands; ‡ Eindhoven Institute of Renewable Energy Systems (EIRES), Eindhoven University of Technology, P.O. Box 513, 5600 MB Eindhoven, The Netherlands

## Abstract

Using potential pulses in Cu-catalyzed CO_2_ electroreduction (CO_2_RR) has recently shown great promise in steering product selectivity. However, the effect of these pulses on the catalyst structure and, therefore, the formation of C_1_ and C_2_ products remain unclear. Herein, we altered the applied potential of the anodic pulse and varied the duration of the cathodic CO_2_RR pulse to investigate the effect of both the oxidation state and restructuring of an oxide-derived Cu nanoparticle catalyst on the CO_2_RR. Shorter cathodic pulses and a higher anodic pulse potential led to a higher ethanol-to-ethylene Faradaic efficiency (FE) ratio, an increase in the C_1_ FE, and a suppression of the hydrogen evolution reaction (HER). We tracked the structural evolution of the CuO catalyst by in situ X-ray absorption spectroscopy (XAS) during the (pulsed) CO_2_RR. The CuO catalyst was fully reduced under potentiostatic conditions, whereas residual CuO_*x*_ was detected during pulsed CO_2_ electrolysis. Quasi-in situ X-ray photoelectron spectroscopy (XPS) further emphasized that the surface of the Cu catalyst was significantly impacted by the application of potential pulses, whereby a higher fraction of CuO_*x*_ was observed with the application of shorter cathodic pulses and higher anodic pulse potentials. Moreover, X-ray diffraction (XRD) and transmission electron microscopy (TEM) showed that sintering and defect formation took place during pulsed CO_2_RR, resulting in an irreversible morphological change, which was associated with the enhanced formation of C_1_ products.

## Graphical abstract

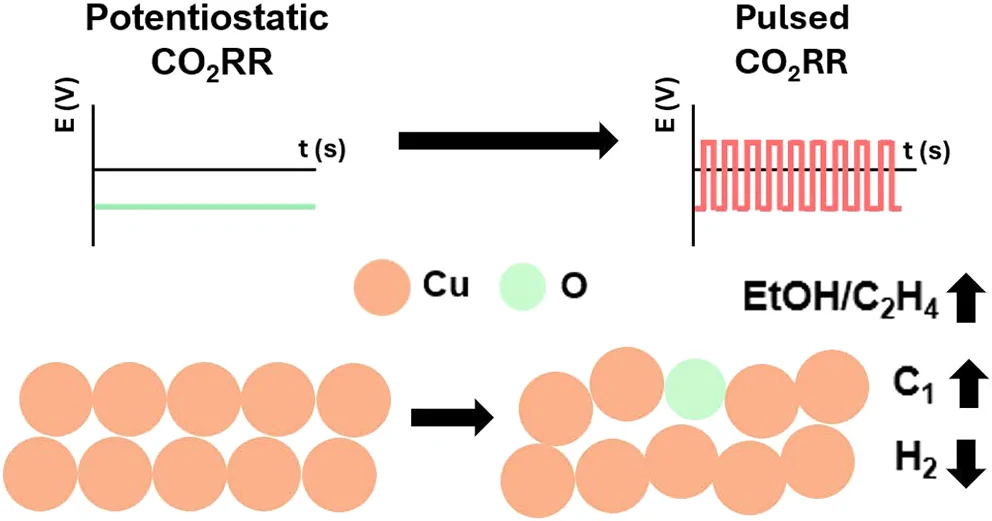

## 1 Introduction

Global climate change is one of the greatest threats humanity is facing, primarily caused by excessive fossil fuel consumption and associated emissions of greenhouse gases like CO_2_.^1^ Therefore, transitioning from fossil energy resources to renewable ones is one of the most significant challenges of this century.^2^ The electrochemical reduction reaction of CO_2_ (CO_2_RR) over copper-based catalysts powered by renewable electricity offers a promising pathway for converting waste CO_2_ into multicarbon products such as ethylene and ethanol.^3^ This approach not only contributes to closing the carbon cycle but also enables the storage of surplus renewable energy in chemical bonds.

Unfortunately, the formation of the desired multicarbon products in Cu-catalyzed CO_2_RR is hindered by the formation of less valuable C_1_ products like CO and CH_4_ and the parasitic hydrogen evolution reaction (HER).^4,5^ Over the past decade, many experimental and theoretical studies have tried to establish structure-performance relationships of Cu-based catalysts for the CO_2_RR with a focus on suppressing the HER and enhancing the formation of C_2_ products.^6,7^ For instance, significant scientific efforts have been devoted to understanding how the crystallographic orientation,^8,9^ defect formation,^10,11^ and the oxidation state of Cu impact the product distribution of CO_2_RR.^12,13^ The presence of interfaces consisting of Cu^+^ and Cu^0^ species was found to play a key role in the formation of C_2_ products.^14^ Such experimental findings were supported by theoretical calculations, which indicated that Cu^+^ sites strongly adsorb CO, followed by subsequent hydrogenation to *CHO.^15^ The work of Sun et al. also found a lower barrier for C–C coupling of *CHO with *CO than dimerization of two *CO. Although stabilization of Cu^+^ species has been frequently reported under CO_2_RR conditions,^16,17^ other reports point out that at the negative potentials used, CuO_*x*_ phases should be fully reduced.^18,19^

A simple and effective way to generate copper oxide species is by applying periodic oxidative potentials during the CO_2_RR, which is commonly termed pulsed electrolysis (PE).^20^ For example, PE applied to a Cu(100) single-crystal electrode revealed an increased selectivity to ethanol, which was linked to the generation of Cu^+^ species during anodic pulses.^21^ A study on oxide-derived copper nanocubes elaborated on the interplay between the formation of copper oxide species and the catalytic activity by changing the pulse lengths during PE.^22^ Also in this case, the coexistence of Cu^+^ and Cu^0^ species was found to enhance ethanol formation, although further oxidation, either induced by shorter cathodic pulses or longer anodic pulses, resulted in the formation of bulk-like copper oxide with enhanced C_1_ product formation. It has also been realized that the formation of oxidized Cu species during pulsed CO_2_RR changes the composition of the surface-adsorbed layer, whereby the surface coverages of H*, OH*, and CO* strongly impact the product distribution.^23,24^ It has been suggested that an increase in the OH coverage, caused by the positive polarization of the electrode during the anodic pulse, results in a decrease in the H coverage during PE.^25^ This results in an increase in the local pH at the electrode, which is associated with a relatively higher coverage of *CO and more favorable C–C coupling kinetics.^26^ By coupling time-resolved in situ Raman measurements with pulsed CO_2_RR on a Cu foil, Li et al. demonstrated that the CuO_*x*_/Cu composition and the population of *CO control the Faradaic efficiency (FE) ratio of C_2_/C_1_.^27^ Other work also emphasized the role of *OH, whereby a narrow window with optimized coverages of *OH and *CO was found to yield more ethanol.^28^ Besides the effect of CuO_*x*_ formation on the adsorbate coverage surface, the application of periodic anodic and cathodic pulses induces irreversible changes to the Cu morphology, which will also alter the product selectivity.^21,29^ For instance, a decrease in the formation of ethylene along with an increase in methane formation has been linked to morphological changes under PE conditions.^22^ In contrast, others mentioned that defects and grain boundaries are formed during pulsed CO_2_RR,^30^ which have been suggested to lead to optimum adsorption energies of *CO and enhanced C_2_ product formation.^31,32^

Although the benefit of PE toward ethanol formation has already been shown for single crystals, well-defined Cu_2_O nanocubes, and Cu foils, these model systems are less attractive for practical applications. Moreover, many of these studies did not investigate the changes in the catalyst morphology during the pulsed CO_2_RR. Therefore, we systematically investigated the impact of pulsed CO_2_RR on an oxide-derived Cu catalyst with a high surface area prepared by flame spray pyrolysis (FSP). In situ X-ray absorption spectroscopy (XAS) and quasi-in situ X-ray photoelectron spectroscopy (XPS) were employed to determine the oxidation states of the bulk and the surface of the Cu catalyst. In particular, we demonstrate that the fractions of Cu^+^ and Cu^0^ species can be controlled by the application of potential pulses with varying time lengths, leading to a higher ethanol-to-ethylene FE ratio when compared to potentiostatic conditions. However, significant irreversible restructuring, including Cu sintering and surface defect formation, was observed after the pulsed CO_2_RR by X-ray diffraction (XRD) and transmission electron microscopy (TEM). To explore the impact of restructuring caused by potential pulses on the catalytic performance, we conducted potentiostatic measurements on an electrode that had undergone a PE. The ethanol-to-ethylene FE ratio returned to similar values with respect to the catalyst that was subjected to only potentiostatic conditions, highlighting the importance of the coexistence of Cu^+^ and Cu^0^ species for ethanol formation. Furthermore, the Faradaic efficiency (FE) for C_1_ products increased, while the HER was suppressed, which can be attributed to defect formation. This work highlights the pivotal role of (in situ) characterization techniques in tracking the structural evolution of the catalysts. It demonstrates how surface reconstruction of Cu catalysts, through the introduction of Cu^+^ species and defect formation, profoundly impacts the product distribution and catalytic performance.

## 2 Experimental Procedures

### 2.1 Catalyst and Electrode Preparation

The CuO nanoparticles were synthesized by flame spray pyrolysis (FSP) following a procedure reported by our group.^33^ The FSP synthesis was performed with a commercial TETHIS NPS10 apparatus. Cu­(NO_3_)_2_·3H_2_O (99%, Acros Organics) was dissolved in absolute ethanol (extra-dry, Biosolve). The Cu concentration was 0.15 M. The mixture was stirred at room temperature until full dissolution of the salt. The solution was injected into the nozzle (5 mL/min) into a methane-oxygen flame (1.5 L/min methane and 3.0 L/min oxygen). The pressure drop at the nozzle was kept at ∼2.5 bar. The formed nanoparticles were recovered from a quartz filter with a plastic spatula and sieved (450 μm sieve) to remove residual quartz fibers. Subsequently, 7.5 mg of the resulting CuO powder was dispersed in 975 μL of isopropanol (99.9%, Sigma-Aldrich) and 25 μL of Nafion (5 wt %, Alfa Aesar). The resulting ink was sonicated for 30 min and drop-cast onto a polished glassy carbon electrode to obtain a catalyst loading of 0.5 mg/cm^2^. The electrodes were dried in air.

### 2.2 Electrochemical Measurements

The glassware for the electrochemical measurements was cleaned by storing overnight in an aqueous solution of 1 g L^–1^ KMnO_4_ (98%, Alfa Aesar) and 0.5 M H_2_SO_4_ (95–97%, Merck). The solution was drained back into the container, and residual stains of KMnO_4_ were removed with a 10% H_2_O_2_ (33%, VWR) solution. The glassware was boiled three times to remove the inorganic impurities.

A Metrohm Autolab PGSTAT302N instrument was used for the catalytic activity measurements. A gastight home-built cell was used for the CO_2_RR experiments. The anodic and cathodic compartments were separated by an anion exchange membrane (Fumasep FAA-3-PK-130). A platinum mesh and a Ag/AgCl electrode (3 M KCl, redox.me) were used as counter and reference electrodes, respectively. Each compartment contained 60 mL of a CO_2_-saturated 0.1 M KHCO_3_ electrolyte (analysis grade, Merck). Prior to the catalytic test, a prereduction step was performed by linear sweep voltammetry from +0.6 V vs RHE to −1.1 V vs RHE (scan rate: 5 mV/s). For the chronoamperometry measurements, the potential was fixed at −1.1 V vs RHE for 1 h. For the pulsed electrolysis measurements, the potential was alternated between *E*_a_ = +0.6 and +0.8 V vs RHE for 1 s and *E*_c_ = −1.1 V vs RHE for 1, 5, or 9 s. The CO_2_ flow rate was kept constant at 15 mL/min.

The Ag/AgCl potentials were converted to RHE and *iR*-corrected by applying the following formula

1 E(VvsRHE)=E(VvsAg/AgCl)+0.21V+0.059×pH−0.85×iR

The value for *iR* was determined by electrochemical impedance spectroscopy with a frequency range of 1 Hz to 10 kHz. A fresh electrode was used for every single measurement, and each potential was measured three times to determine the error bars. The electrochemical surface area (ECSA) was determined by the double-layer capacitance method. A non-Faradaic potential range was selected to conduct cyclic voltammetry (CV) at scan rates of 10, 25, 50, 75, and 100 mV s^–1^ after CO_2_RR in CO_2_-saturated 0.1 M KHCO_3_ supporting electrolyte.

### 2.3 Catalyst Characterization

#### 2.3.1 X-ray Diffraction (XRD)

The X-ray diffractograms were acquired with a Bruker D2 Phaser diffractometer with a Cu Kα (0.15406 nm) source. An increment size of 0.01° and a time of 0.75 s were used in the 2θ range from 30 to 65°.

#### 2.3.2 Transmission Electron Microscopy (TEM)

Transmission electron microscopy (TEM) was performed with an FEI Tecnai (Sphera) instrument. The acceleration voltage was set at 200 kV. The samples were prepared by coating a Cu grid with the catalyst dispersed in ethanol before and after the reaction. The particle size of the CuO precursor was determined with ImageJ software.

#### 2.3.3 X-ray Photoelectron Spectroscopy (XPS)

XPS spectra of the as-prepared powder sample were acquired using a K-α ultrahigh-vacuum X-ray photoelectron spectrometer (Thermo Fisher Scientific) with a monochromatic Al anode (Kα = 1486.6 eV, 72 W) X-ray source with a spot size of 400 μm. A pass energy of 50 eV was used for the measurement. The quasi-in situ XPS measurements were performed on a SPECS Phoibos NAP-150 electron analyzer with a SPECS electrochemical cell. The core-line spectra were acquired using a monochromatic Al anode (Kα = 1486.6 eV, SPECS XR-50) operated at 50 W. The pass energy was set to 40 eV with a step size of 0.1 eV and a dwell time of 0.5 s. Survey, Cu 2p, Cu LMM, O 1s, and C 1s spectral lines were recorded for all samples. The binding energy was corrected by setting the binding energy of the C–C component of the C 1s spectra to 284.8 eV. An IviumStat potentiostat was used for electrochemical measurements. The working electrode was a carbon paper (Sigracet 22BB) with a catalyst loading of 0.5 mg cm^–2^. A Pt foil and a reversible hydrogen electrode (RHE, Mini-HydroFlex, Gaskatel) were used as the counter and reference electrodes, respectively. A 0.1 M CO_2_-saturated KHCO_3_ solution was used as the electrolyte. For the chronoamperometry measurements, the potential was kept constant at −1.1 V vs RHE for 1 h. For the pulsed electrolysis experiments, the sequence consisted of a cathodic pulse of −1.1 V vs RHE for 1, 5, or 9 s and an anodic pulse (+0.6 or +0.8 V vs RHE) for 1 s. The total measurement time was 1 h. To prevent oxidation by air, a continuous flow of Ar was purged through the buffer chamber and the electrochemical cell during the electrochemical experiments and sample transfer. The electrode and cell were washed with Ar-purged ultrapure water to remove salt residue and transferred to the ultrahigh-vacuum (UHV) chamber. The XPS spectra were fitted with the CasaXPS software (version 2.3.23). Values from the literature were used to fit the Cu species.^34^

#### 2.3.4 X-ray Absorption Spectroscopy (XAS)

The XAS data were collected at the ROCK beamline of the SOLEIL synchrotron facility in Paris, France. A Si(111) monochromator was used for the energy selection. A custom-made PEEK electrochemical cell was used for in situ measurements. The cell geometry was adapted to meet the requirements of synchrotron measurements while maintaining electrochemical conditions as close as possible to those employed in the H-cell used for product quantification. The measurements were performed at the Cu K-edge (8979 eV) in the fluorescence mode with a PIPS detector. The working electrode was a carbon paper substrate (Sigracet 22B) with a catalyst loading of 0.5 mg cm^–2^. The back of the carbon paper faced a Kapton window. A Pt mesh and reversible hydrogen electrode (RHE, Mini-HydroFlex, Gaskatel) were used as counter and reference electrodes, respectively. An anion exchange membrane (Fumasep FAA-3-PK-130) was used to separate the anodic and cathodic compartments. An IviumStat potentiostat was used to control the potential. A 0.1 M CO_2_-saturated KHCO_3_ solution was circulated through the cell at a flow rate of 5 mL/min. The chronoamperometry measurements were conducted at −1.1 V vs RHE for 1 h. For the pulsed electrolysis experiments, the sequence consisted of a cathodic pulse of −1.1 V vs RHE for 1, 5, or 9 s and an anodic pulse (+0.6 or +0.8 V vs RHE) for 1 s. The total measurement time was 1 h. For EXAFS data analysis, the *k*-weighting was 2, and the *k*-range was from 3 to 10. Athena and Artemis software were used for data treatment. The amplitude reduction factor S_0_^2^ was determined by fitting the Cu–O and Cu–Cu single scattering paths of the first shell of CuO and Cu foil, respectively.

### 2.4 Product Analysis

The formed gaseous products were analyzed by online gas chromatography (GC Trace 1300—Thermo Fisher Scientific). A TCD (Hayesep Q precolumn and Shin-Carbon ST column) and an FID (Al_2_O_3_/KCl column), equipped with a methanizer, were used for the detection of H_2_ and CO_2_RR products, respectively. The liquid products were analyzed by ^1^H nuclear magnetic resonance spectroscopy (^1^H NMR) using the water suppression mode on a Bruker 400 MHz instrument. A solution of 10 mM dimethyl sulfoxide (DMSO) (99%, Biosolve) and 50 μM phenol (99%, Merck) in D_2_O (99.9%, Merck) was used as the internal standard. The Faradaic efficiencies were calculated with the following formula

2 FE=z×n×F/Q

with *z* the number of electrons involved in the formation of the product, *n* the number of moles, *F* the Faraday constant (96,485 C mol^–1^), and *Q* the total charge. For pulsed electrolysis experiments, only the cathodic charge was considered to calculate the Faradaic efficiencies.

## 3 Results and Discussion

The polycrystalline CuO nanoparticles used in this study were prepared by FSP. A 0.15 M solution of Cu­(NO_3_)_2_·3H_2_O in ethanol was injected into a flame through the nozzle of the FSP apparatus. Evaporation and combustion of the organics led to the formation of small CuO nanoparticles. According to TEM, the average size of the CuO nanoparticles was 12 nm. This is in good agreement with the average crystallite size of 13 nm determined by Scherrer analysis of the XRD pattern, which indicates exclusive formation of CuO. The physicochemical characterization of the fresh and used catalysts will be discussed below. The Cu nanoparticles were immobilized on a glassy carbon electrode for electrochemical characterization by cyclic voltammetry (CV) and catalytic performance tests.

CVs recorded in Ar- and CO_2_-saturated 0.1 M KHCO_3_ electrolyte are shown in Figure 1. We used a potential window from −0.9 to +1.3 V vs RHE and a scan rate of 50 mV/s for these CVs. In the CV using Ar-saturated 0.1 M KHCO_3_, the peaks A at +0.6 V vs RHE and B at +0.8 V vs RHE correspond to the formation of Cu^+^ and Cu^2+^, while peaks B′ and A′ could be assigned to the reduction of Cu^2+^ to Cu^+^ and the subsequent reduction to metallic Cu, respectively.^21,35,36^ At more negative potentials, the current increase is due to hydrogen evolution. In CO_2_-saturated 0.1 M KHCO_3_ electrolyte, the current densities are significantly higher between −0.3 and −0.9 V vs RHE, implying that the catalyst is active for the CO_2_RR. The intense peak in the potential window of +0.5 to +1.2 V corresponds to the formation of CuO_*x*_ and oxidation of in situ-formed CO, which is one of the products of the CO_2_RR.^37^ The reduction peaks A′ and B′ are also visible for the CV recorded in CO_2_-saturated 0.1 M KHCO_3_.

**Figure 1:**
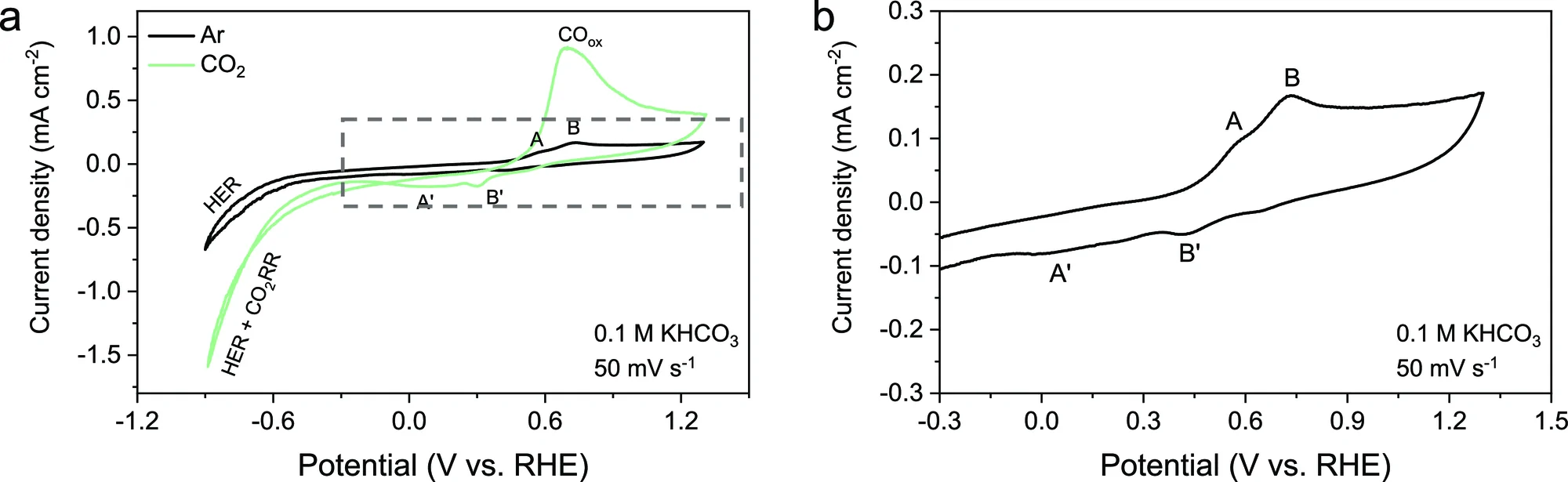
(a) Cyclic voltammograms of the CuO catalysts in 0.1 M KHCO_3_ in Ar (black) and CO_2_ (green). The area indicated with the gray dashed lines is shown in more detail in (b). A scan rate of 50 mV/s was used.

Based on the CVs, we selected +0.6 and +0.8 V vs RHE (formation of Cu^+^ and Cu^2+^, respectively) as potentials for the anodic pulse (*E*_a_). The corresponding measurements will be denoted as PE +0.6 V and PE +0.8 V. The time of the anodic pulse (*t*_a_) in this study was always 1 s. For the cathodic pulse (*E*_c_), a potential of −1.1 V vs RHE was chosen, as this potential has been demonstrated to result in high FEs for C_2_ products in Cu-catalyzed CO_2_RR.^38,39^ The duration of the cathodic pulse (*t*_c_) was altered to 1, 5, and 9 s, and the total time of the (pulsed) CO_2_RR measurements was 1 h. Typical profiles of the current over time during (pulsed) CO_2_RR are given in Figures S1 and S2. Figure 2a shows the FEs of the oxide-derived Cu catalyst at a constant potential of −1.1 V vs RHE (no pulse). FEs of roughly 42% for H_2_, 6% for C_1_ products (CO, methane, and formate), and 35% for C_2_ products (ethylene, ethanol, and acetate) were observed. This product distribution is typical for Cu electrodes during the CO_2_RR under potentiostatic conditions.^40^ As shown in Figure 2b–d, the FEs were significantly impacted by the periodic application of pulses. Shorter cathodic pulses led to a decrease in the total FE to reaction products. The same holds true when the total FE of PE +0.8 V measurements is compared to that of the PE +0.6 V ones. This decrease can be attributed to the consumption of electrons for the reduction of the partially oxidized surface obtained during the anodic pulse. Oxidation of CO_2_RR products during the anodic pulse may also contribute to the lower FE to reaction products. Furthermore, we observed a decrease in the relative formation of H_2_ versus CO_2_RR products for higher *E*_a_ (+0.8 vs +0.6 V) and shorter *t*_c_ (1 s compared to 5 or 9 s) during PE. It has been suggested that OH* plays an important role in the product distribution during pulsed CO_2_RR.^25^ During the anodic pulse, OH^–^ species adsorbed onto the surface during the positive polarization of the electrode might result in a higher local pH near the electrode surface. Subsequently, the accumulation of CO* and OH* at the surface results in suppressed H adsorption and, therefore, the HER during the cathodic pulse.^25^ In a study by Herzog et al., it was found that the duration of the anodic pulse substantially affected the product distribution.^28^ In another work, it was suggested that a longer anodic pulse can deplete OH^–^ species due to significant formation of Cu_2_O from Cu, resulting in a decrease in the local pH, facilitating C_1_ product formation during the cathodic pulse.^30^ In line with this, at a *t*_c_ of 1 s, which is the shortest with respect to the constant anodic pulse length, we observed an increase in the FE toward CO and methane. Under such conditions, we expect that the surface cannot be fully reduced again, which is reflected in the product distribution. Enhanced C_1_ product formation has been associated with the presence of bulk-like copper oxide.^22^ The ethanol-to-ethylene FE ratios obtained during PE measurements are shown in Figure 2e. Under potentiostatic conditions, this ratio was ∼0.7. Similar values were obtained for PE +0.6 V and PE +0.8 V for *t*_c_ = 9 s. The ethanol-to-ethylene FE ratio increased slightly to 0.75 for PE +0.6 V and *t*_c_ = 5 s. We hypothesize that the long *t*_c_ of 9 s or the lower *E*_a_ of +0.6 V for *t*_c_ = 5 s leads to a surface state during the cathodic pulse that is very similar to the surface state during the potentiostatic measurement. By increasing *E*_a_ to +0.8 V or by decreasing the *t*_c_ to 1 s, we observed a drastic increase in the ethanol-to-ethylene FE ratio up to almost 2.5. The higher anodic potential will increase the Cu oxidation degree. We can expect that a higher fraction of oxidized Cu species is maintained during the cathodic reaction, which can explain the enhanced formation of ethanol.^21^ This interpretation is supported by the average anodic and cathodic pulse charges per cycle (Figure S3), which show an increase in the anodic-to-cathodic charge ratio (*Q*_a_/|*Q*_c_|) with decreasing *t*_c_, consistent with a stronger influence of oxidative surface processes during PE. However, it is important to note that the FE toward ethylene decreased with increasing frequency of the anodic pulses (shorter *t*_c_), which led to a strong increase in the FE of CO and CH_4_. This is most likely due to the extensive oxidation and possibly restructuring of the Cu surface.^22^ The electrochemical surface area (ECSA) after the (pulsed) CO_2_RR, measured by the double-layer capacitance method (Figures S4–S8 and Table S1), was found to substantially decrease, up to 6 times during the pulsed measurements as compared to the potentiostatic measurement. This indicates severe restructuring and likely sintering of the catalyst. The impact of pulsing is also reflected in the partial current densities (Figure S9). While the geometric partial current densities for both ethylene and ethanol decreased during PE, normalization by the ECSA revealed an increase in the intrinsic activity toward these C_2_ products. The highest ECSA-normalized partial current densities for ethylene and ethanol were found for an intermediate pulse protocol with *t*_c_ = 5 s. Under more aggressive pulsing conditions (*t*_c_ = 1 s), the ECSA-normalized partial current density for ethylene decreased more strongly than that for ethanol, consistent with the observed increase in the ethanol-to-ethylene FE ratio.

**Figure 2:**
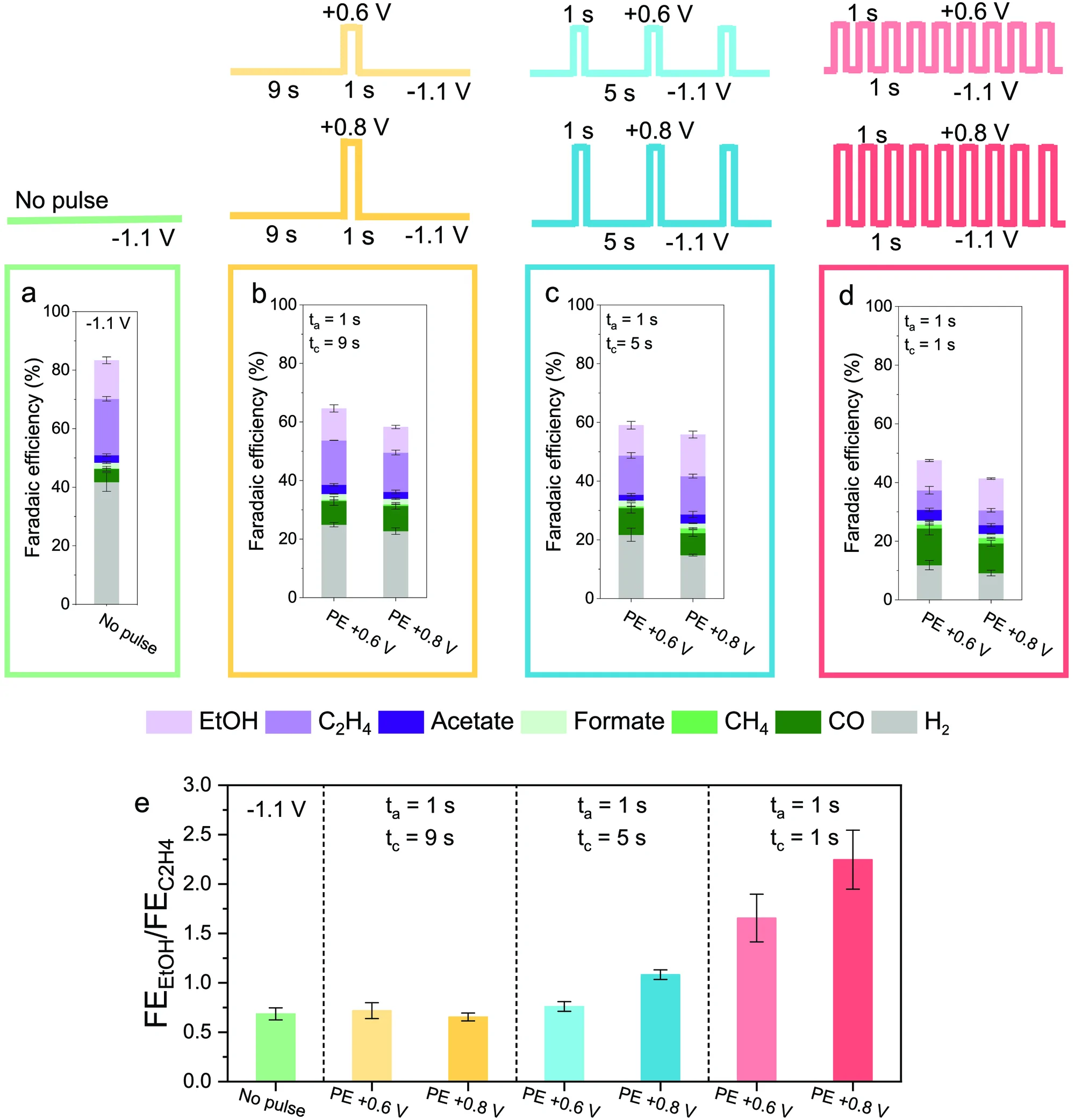
Potential profiles and Faradaic efficiencies for chronoamperometry at (a) −1.1 V and during PE (*E*_c_ = −1.1 V, *E*_a_ = +0.6 or +0.8 V, and *t*_a_ = 1 s) with *t*_c_ = (b) 9 s, (c) 5 s, and (d) 1 s. (e) Selectivity ratio of ethanol-to-ethylene for the conducted experiments.

The structural evolution of the CuO catalyst was studied before and after (pulsed) CO_2_RR by ex situ XRD (Figure 3). The broad background signal between 40 and 55° originates from the glassy carbon support. The diffraction pattern of the as-prepared sample in Figure 3a exhibited reflections characteristic of monoclinic CuO at 35.8° (200) and 38.9° (111).^41^ The CuO crystallite size, determined using the Scherrer equation applied to the CuO (111) reflection, was found to be ∼13 nm. After the (pulsed) CO_2_RR, the CuO catalyst was reduced during all electrochemical measurements, as evidenced by the disappearance of these reflections. This was accompanied by the appearance of the Cu_2_O­(111) reflection at 36.5° and strong metallic reflections of Cu(111) at 43.4° and Cu(200) at 50.5°. As these XRD patterns were obtained after air exposure, the presence of Cu_2_O might be the result of oxidation of metallic Cu by air. The Cu crystallite sizes, estimated by applying the Scherrer equation to the Cu(200) reflection, were approximately 14, 18, and 19 nm for the nonpulsed, 9–5 s pulsed, and 1 s pulsed samples, respectively. This increase in average particle size indicates that the Cu particles have sintered during the (pulsed) CO_2_RR conditions. Moreover, the sintering is stronger with increasing frequency of anodic pulses. Similar sintering behavior of small Cu nanoparticles has been observed in other studies on Cu-based catalysts for the electrochemical reduction of CO (CORR) and CO_2_RR.^42,43^

**Figure 3:**
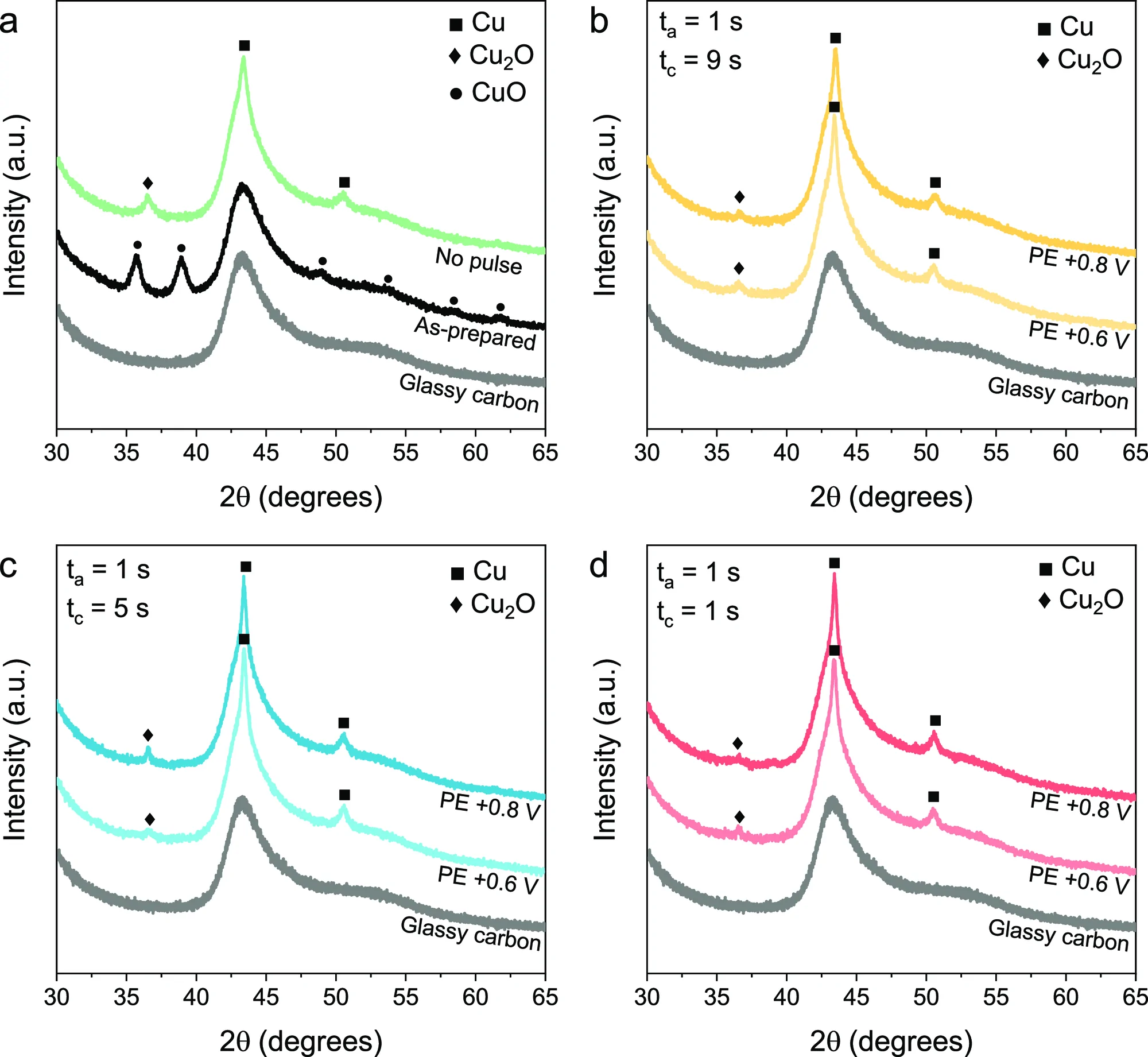
Ex situ XRD of (a) the as-prepared sample and after the CA at −1.1 V vs RHE (no pulse) and after pulsed CO_2_RR (with *E*_a_ = +0.6 or +0.8 V vs RHE and *t*_a_ = 1 s) with (b) *t*_c_ = 9 s, (c) *t*_c_ = 5 s, and (d) *t*_c_ = 1 s.

TEM was used to study the structural evolution of the CuO catalyst before and after (pulsed) CO_2_RR (Figures 4, S10, and S12). The average particle size of the as-prepared sample was found to be 11.6 ± 1.9 nm (Figure S11). However, exposure to (pulsed) CO_2_RR resulted in a significantly broadened particle size distribution. Postreaction TEM analysis revealed the coexistence of very small particles only a few nm in size, medium-sized particles (∼30 nm), and large agglomerates. Besides, amorphous Cu­(OH)_*x*_ can be formed during PE, which cannot be identified by XRD.^44^ Although a detailed particle size analysis could not be obtained after reaction due to particle agglomeration, the TEM observations are consistent with increasing crystallite size during PE as determined by XRD and the pronounced decrease in ECSA, both indicating significant catalyst sintering. Detailed inspection of TEM images obtained after PE +0.8 V with a short cathodic pulse *t*_c_ = 1 s (Figure S13) revealed the presence of grain boundaries between the primary particles. The presence of such grain boundaries has been linked to increased CO binding and favorable CO dimerization kinetics.^32,45^ In another work, the grain boundary density was shown to increase under harsh PE conditions.^46^ Figure S14 also shows the presence of very small nanoparticles with a size of only a few nm after CO_2_RR. These Cu particles are likely formed through a dissolution and redeposition mechanism.^47,48^

**Figure 4:**
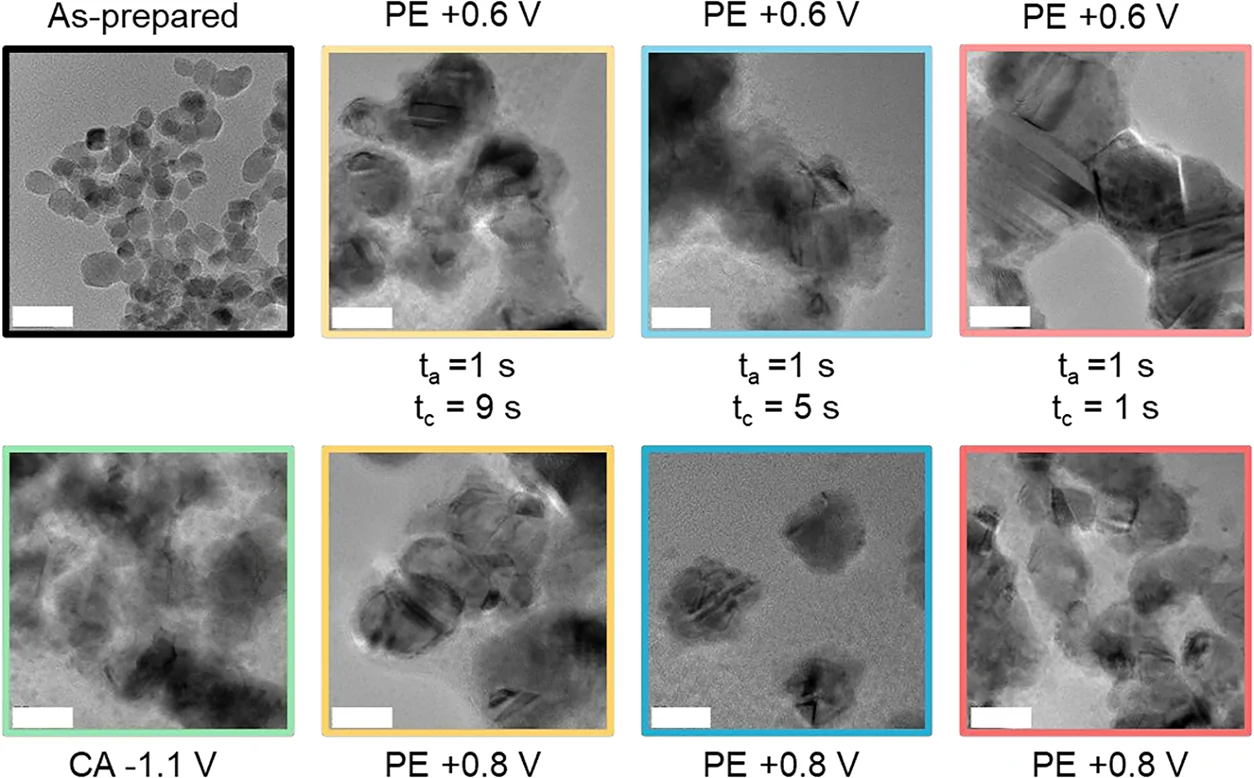
TEM images of the as-prepared CuO catalyst and after the (pulsed) CO_2_RR measurements. The scale bars correspond to 30 nm.

To track the changes in the oxide-derived Cu catalyst during the (pulsed) CO_2_RR, we performed in situ XAS measurements. The normalized Cu K-edge X-ray absorption near-edge structure (XANES) spectra during the CA at −1.1 V vs RHE (no pulse) are shown in Figure 5a. A comparison of the as-prepared sample (*t* = 0 min) with the references reveals that the catalyst is initially present as CuO, consistent with the XRD results. Under potentiostatic conditions, the catalyst rapidly reduced, and the XANES spectrum resembled the Cu foil reference after 60 min. During pulsed electrolysis, the majority of CuO in the initial catalyst reduced to metallic Cu (Figure S15). Although the XANES spectra remained dominated by features characteristic of metallic Cu under all investigated conditions, relative changes in the Cu oxidation state could be quantified from the ΔXANES spectra and their integrated area. The time resolution of these measurements did not allow us to track the evolution of XANES features during individual potential pulses. Still, we observed clear differences in the oxidation state by comparing ΔXANES of averaged spectra recorded during the last 10 min of the measurements, whereby the average spectrum of the PE measurement was subtracted from the spectrum of the CA measurement (Figure 5b,c). The pre-edge peak of metallic Cu at 8980 eV decreased in intensity during the PE measurements. The increase of the feature at 9000 eV can be attributed to the presence of oxidized Cu species. These changes increased when the cathodic pulses were shorter (smaller *t*_c_) and for more positive anodic pulse potentials (*E*_a_). The differences between the PE +0.6 V and PE +0.8 V measurements for different cathodic pulse durations are further emphasized by the integrated area based on the absolute ΔXANES values between 8970 and 9005 eV (Figure 5d,e). This integrated ΔXANES area provides a quantitative measure of the relative oxidation state changes and shows that shorter cathodic pulse durations and more positive anodic pulse potentials lead to a higher degree of Cu oxidation.

**Figure 5:**
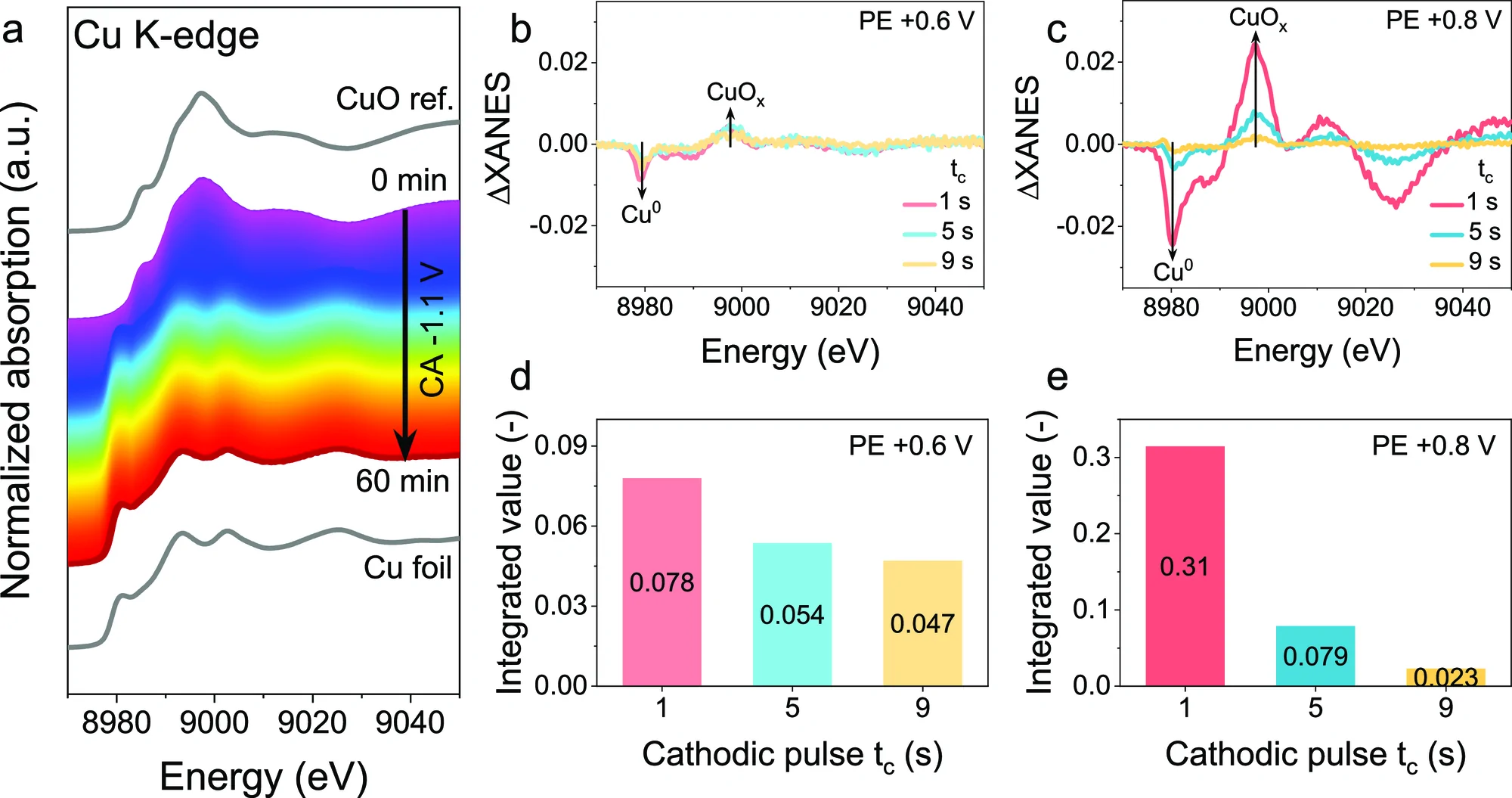
(a) Normalized Cu K-edge XANES spectra of the CuO catalyst during the chronoamperometry for 1 h at −1.1 V vs RHE (no pulse) in CO_2_-saturated 0.1 M KHCO_3_ electrolyte. Changes in the XANES as compared to the reduced sample after the CA at different cathodic pulse durations, *t*_c_, for (b) PE +0.6 V and (c) PE +0.8 V. Absolute values of the integrated area between 8970 and 9005 eV of the ΔXANES for (d) PE +0.6 V and (e) PE +0.8 V. The duration of the anodic pulse, *t*_a_, was 1 s, and the cathodic pulse potential, *E*_c_, was −1.1 V vs RHE during the PE measurements.

The coordination environment around the Cu atoms before (pulsed) CO_2_RR and under reaction conditions was determined by Fourier-transformed extended X-ray absorption fine structure (FT-EXAFS). The as-prepared sample is predominantly in the Cu­(II) state, in line with the XANES analysis. The Cu–O and Cu–Cu contributions at ca. 1.5 and 2.5 Å, respectively, for the as-prepared sample (Figure 6a) are characteristic of Cu^2+^ in an oxidic environment as in CuO.^49^ The FT-EXAFS during the CA at −1.1 V vs RHE (Figure 6b) shows a new peak at 2.2 Å, corresponding to Cu–Cu bonds in metallic Cu.^50^ Similar FT-EXAFS were obtained for the PE experiments (Figure 6c–e), consistent with the XANES analysis. The EXAFS data obtained during the last 10 min of the CO_2_RR were fitted (Table S2). The resulting coordination number (CN) of the metallic Cu–Cu shell of about 9 during the CA and PE measurements indicates the presence of Cu particles. For PE + 0.6 V with *t*_c_ = 1 s and PE + 0.8 V with *t*_c_ = 1 and 5 s, the FT-EXAFS fits also included Cu–O scattering paths with relatively small CN of at most 0.3, which indicates that a small fraction of Cu is present in the oxidized form. Thus, the XAS results show how the anodic potential and the duration of the cathodic pulse at fixed anodic pulse length affect the oxidation state of Cu catalyst.

**Figure 6:**
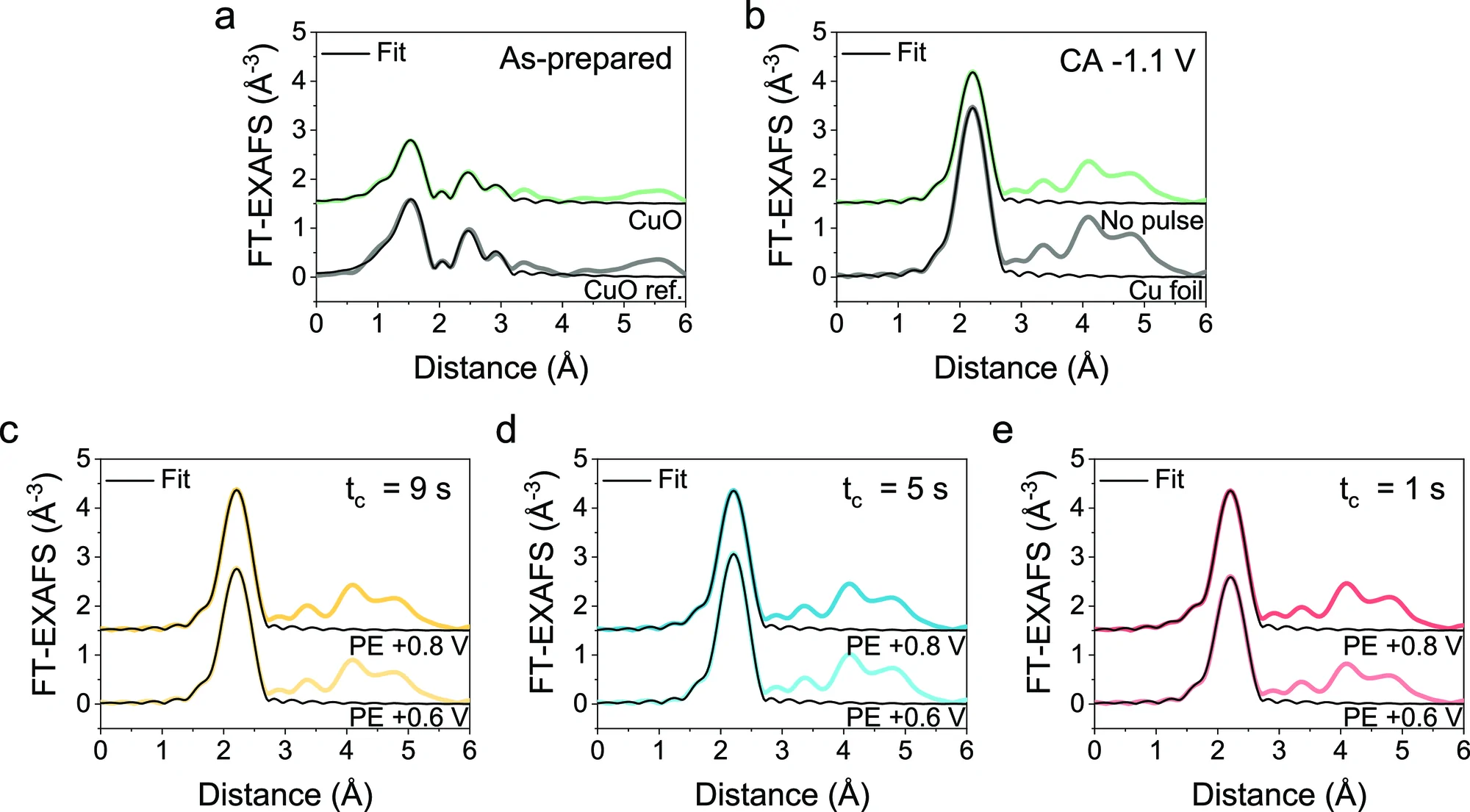
Cu K-edge FT-EXAFS of (a) as-prepared CuO and the CuO reference, (b) during the CA at −1.1 V vs RHE and the Cu foil reference. Cu K-edge FT-EXAFS during the PE +0.6 V and PE +0.8 V and *t*_c_ values of (c) 9 s, (d) 5 s, and (e) 1 s. The duration of the anodic pulse, *t*_a_, was 1 s and the cathodic pulse potential, *E*_c_, was −1.1 V vs RHE during the PE measurements.

To study the oxidation state of the surface Cu species, we employed quasi-in situ XPS after 1 h (pulsed) of the CO_2_RR. The electrochemical measurements were conducted in a CO_2_-saturated 0.1 M KHCO_3_ electrolyte under an inert atmosphere of Ar. It is important to note that the sequence was interrupted after the anodic pulse, typically resulting in a more-oxidized surface than after the cathodic pulse. Therefore, the oxidation states identified by quasi-in situ XPS should be regarded as an upper estimate of the degree of oxidation during PE, as the measurements emphasize the oxidized surface species generated during the anodic pulse. Afterward, the sample was washed with water to remove salt residues and transferred to the XPS analysis chamber without air exposure. The quasi-in situ XPS results are shown in Figure 7. The Cu 2p_3/2_ spectrum of the as-prepared sample shows a peak at a binding energy of 933.7 eV and a satellite feature in the 939–945 eV range characteristic of CuO,^34^ confirming the earlier XRD and XAS findings. After 1 h of CO_2_RR at −1.1 V vs RHE (no pulse), the peak of the Cu 2p_3/2_ line was at a lower binding energy, and the satellite feature had disappeared. The binding energy of 932.6 eV indicates that the surface was reduced to Cu^0^ or Cu^+^ species. This peak was also present after the pulsed CO_2_RR conditions. However, for *t*_c_ = 1 s, the satellite feature emerged together with a shoulder on the high-binding-energy side of the Cu 2p_3/2_ line, indicating the presence of Cu^2+^ species alongside Cu^0/+^. To distinguish between Cu^0^ and Cu^+^ species, the Cu LMM lines were analyzed (Figure 7b). The Cu LMM spectrum for the as-prepared sample showed the exclusive presence of Cu^2+^, in line with the Cu 2p_3/2_ XPS analysis.^34^ The Cu LMM spectrum recorded after 1 h CA at −1.1 V vs RHE shows predominant formation of Cu^0^, indicating that the CuO catalyst was fully reduced under these conditions. A similar result was obtained under pulsed CO_2_RR conditions with an *E*_a_ of +0.6 V and a *t*_c_ of 9 s. When applying a more positive anodic pulse, i.e., *E*_a_ at +0.8 V vs RHE, or by decreasing the cathodic pulse duration, the Cu LMM feature corresponding to Cu^+^ increased in intensity at the expense of Cu^0^. At a short *t*_c_ value of 1 s, the feature associated with Cu^2+^ also appeared. Notably, the surface-sensitive quasi-in situ XPS results revealed greater variations in the oxidation state of Cu compared with the bulk-sensitive in situ XAS measurements, emphasizing that the oxidation of the catalyst predominantly occurs at the surface during the pulsed CO_2_RR. Furthermore, this difference may also arise from the nature of the experiments, as the quasi-in situ XPS measurements probe the catalyst surface immediately after an anodic pulse, whereas the in situ XAS measurements reflect the average catalyst state during the operation. While the coexistence of metallic Cu and CuO_*x*_ species at the surface has been associated with enhanced ethanol formation, deeper oxidation to CuO has been linked to increased C_1_-product formation.^51–53^ This aligns well with our observations on the catalytic activity, such as the ethanol-to-ethylene FE ratio and the formation of C_1_ products.

**Figure 7:**
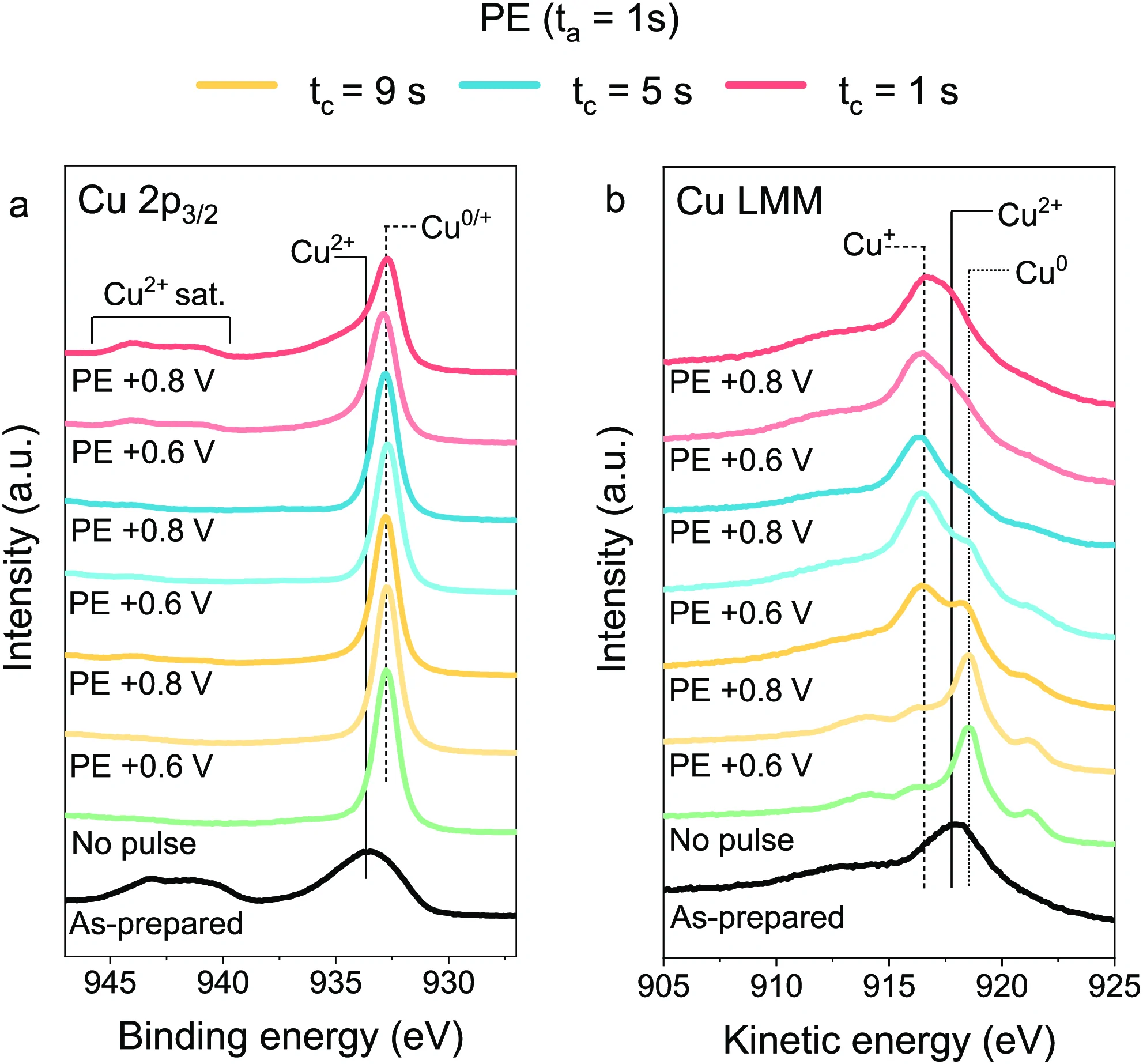
Quasi-in situ XPS spectra: (a) Cu 2p_3/2_ and (b) Cu LMM lines of the CuO precursor before and after (pulsed) CO_2_RR.

To address whether the changes in the ethanol-to-ethylene FE ratio originate from the presence of oxidized Cu species during the cathodic reactions or by severe restructuring of the catalyst during pulsed CO_2_RR, we conducted a 1 h CA measurement at −1.1 V vs RHE (no pulse) on an electrode that was pretreated for 1 h with pulses with *E*_a_ = +0.8 V vs RHE and *t*_a_ = *t*_c_ = 1 s (Figure 8). It is reasonable to assume that the CA of 1 h will reduce residual Cu-oxide species obtained during the PE conditions. Figure 8a shows the FE results of the CA at −1.1 V vs RHE after the pulse pretreatment. The FEs for the CA at −1.1 V vs RHE without pulse pretreatment and for PE +0.8 V with *t*_a_ = *t*_c_ = 1 s are shown again. The lower total FE most likely originates from the consumption of the current for the reduction of the catalyst after the pulse pretreatment procedure. Figure 8b shows that the ethanol-to-ethylene FE ratio during this experiment returns to its initial value of ∼0.7 obtained under potentiostatic conditions (no pulse). Thus, it is likely that the changes in the product distribution are caused by the presence of oxidized Cu species obtained during the short anodic pulses. However, some differences were observed in the FE toward H_2_ and C_1_ products for the CA at −1.1 V vs RHE with and without pulse pretreatment. During the CA after the pulse pretreatment, the H_2_ FE decreased from 42 to 28%, while the CO FE increased from 5 to 10%, and a small amount of CH_4_ was observed (ca. 1%). The ECSA for the pulse-pretreated electrodes after the 1 h CA at −1.1 V was similar to those obtained after the PE +0.8 V measurement with *t*_a_ = *t*_c_ = 1 s (Figures 8c, S4b, and S6) and significantly lower than for the electrode that was only subjected to potentiostatic conditions. The lower current during the chronoamperometry after pulse pretreatment can be explained by this decrease in ECSA (Figure S1). Based on this, we propose that enhanced formation of C_2_ products is linked to an optimal balance between Cu^0^ and Cu^+^ species. For a fully metallic Cu catalyst, the HER dominates, whereas an excessive presence of oxidized Cu species leads to a shift toward C_1_ products at the expense of ethylene formation. The oxidized Cu species can be generated by applying potential pulses in the oxidation regime of Cu, with the applied potential and pulse duration serving as key parameters to regulate the fraction of Cu^+^ species. However, the application of potential pulses inevitably induces irreversible morphological changes alongside the formation of oxidized Cu species. Our TEM results clearly demonstrated the formation of defects, such as grain boundaries. While the recovery of the ethanol-to-ethylene FE ratio during the subsequent chronoamperometric experiment suggests that the presence of oxidized Cu species and the resulting Cu^+^/Cu^0^ balance play an important role in determining the C_2_ selectivity, the persistent differences in H_2_ and C_1_ product formation indicate that irreversible catalyst restructuring may also contribute to the observed catalytic behavior. However, the present results do not allow us to unambiguously distinguish the influence of these defects from other consequences of restructuring, such as changes in surface area or morphology. Furthermore, it should be noted that decreasing the cathodic pulse duration simultaneously increases the frequency of pulse sequence and the cumulative exposure of the catalyst to anodic conditions. Therefore, care should be taken not to attribute the observed changes solely to the cathodic or anodic pulse. Overall, these results suggest that both chemical and structural modifications contribute to the catalytic performance during CO_2_RR.

**Figure 8:**
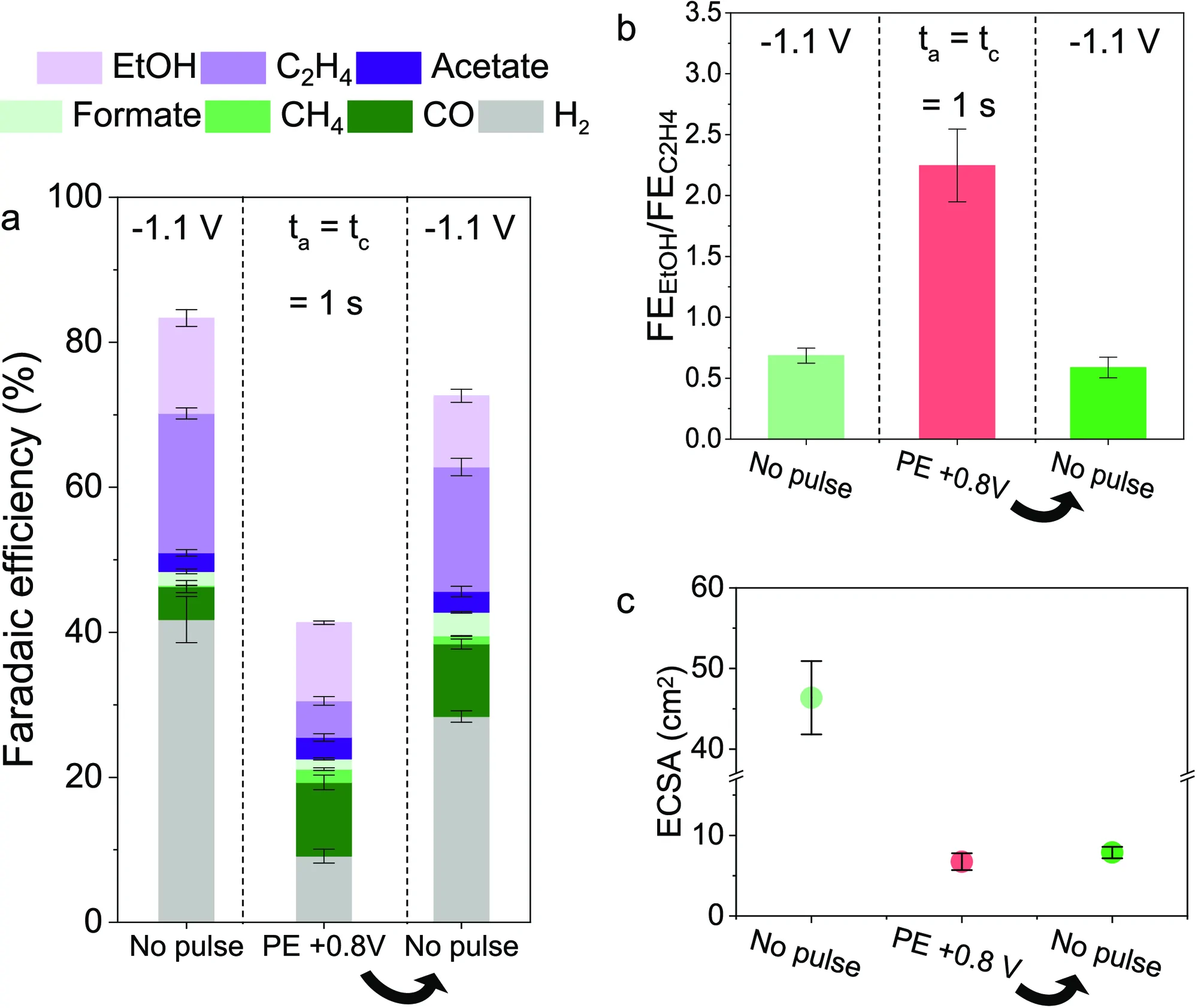
(a) Faradaic efficiencies, (b) ethanol/ethylene selectivity ratio, and (c) the ECSA under potentiostatic (−1.1 V vs RHE) or pulsed (*E*_a_ = +0.8 V, *E*_c_ = −1.1 V and *t*_a_ = *t*_c_ = 1 s) conditions. The arrows indicate that the electrode subjected to PE conditions was subsequently measured at constant potential (no pulse).

## 4 Conclusions

We explored the effect of the anodic pulse potential and cathodic pulse length on the restructuring of oxide-derived Cu catalysts and their selectivity during the CO_2_RR. Our results demonstrate that the ethanol-to-ethylene FE ratio can be readily adjusted, whereby a higher anodic pulse potential (+0.8 vs +0.6 V) and shorter cathodic pulses (1 s instead of 5 or 9 s) typically led to an increase in this selectivity ratio. In situ XAS measurements revealed that the majority of the bulk of the CuO precursor was reduced to metallic Cu during (pulsed) CO_2_RR, while subtle increases in oxidation state were observed with increasing anodic pulse potential and pulse frequencies. Quasi-in situ XPS clearly demonstrated a systematic increase of CuO_*x*_ species on the nanoparticle surface by the application of higher anodic pulse potentials and a shorter cathodic pulse length. Furthermore, a substantial loss of the ECSA was measured when applying PE conditions as compared to the potentiostatic conditions, while XRD and EXAFS results indicated only minor increases in Cu (crystallite) size. Here, TEM measurements showed that significant defect formation, such as grain boundaries, and sintering took place during pulsed CO_2_RR, providing an explanation for the ECSA, XRD, and EXAFS results. Testing the CO_2_RR performance at constant potential after harsh PE conditions revealed that the increase in C_2_ production and ethanol-to-ethylene ratio could be reversed, while the CO, CH_4_, and H_2_ formation rates remained high. Hence, the increase in the ethanol selectivity observed during PE appears to be primarily associated with the presence of residual copper oxide species. In contrast, the persistent changes in H_2_ and C_1_ product formation after pulse pretreatment suggest a contribution from irreversible catalyst restructuring. These findings provide valuable insights into the effect of restructuring on different products during electrochemical CO_2_ conversion and highlight the importance of the multitechnique approach to identify structure–activity relationships.

## Supplementary Material



## Notes

Examples of current profiles and ECSA measurements; TEM images; and XAS data and fitting parameters (PDF)
