# Supplementary material for: Impact of Pulsed CO2 Electrolysis on Surface Structures and Product Distribution for Oxide-Derived Cu
Source: J Phys Chem C Nanomater Interfaces. 2026 Oct 19;130(43):14911–22. doi: 10.1021/acs.jpcc.6c05013 (PMC13726460; doi:10.1021/acs.jpcc.6c05013)
Supplement: Supplementary file 1 [file jp6c05013_si_001.pdf]

Supporting Information

Impact of Pulsed CO_2 Electrolysis on Surface Structures and Product Distribution for Oxide-Derived Cu

Bianca Ligt,[1] Jérôme F.M. Simons,[1] Jason M.J.J. Heinrichs,[1] Laura B. Donk,[1] Marta Costa Figueiredo,[1,2] Emiel J.M. Hensen[1]

[1] Laboratory of Inorganic Materials and Catalysis, Department of Chemical Engineering and Chemistry, Eindhoven University of Technology, P.O. Box 513, 5600 MB Eindhoven, The Netherlands

[2] Eindhoven Institute of Renewable Energy Systems (EIRES), Eindhoven University of Technology, P.O. Box 513, 5600 MB Eindhoven, The Netherlands

Corresponding author: Emiel J.M. Hensen, e.j.m.hensen@tue.nl

Supplementary Figures

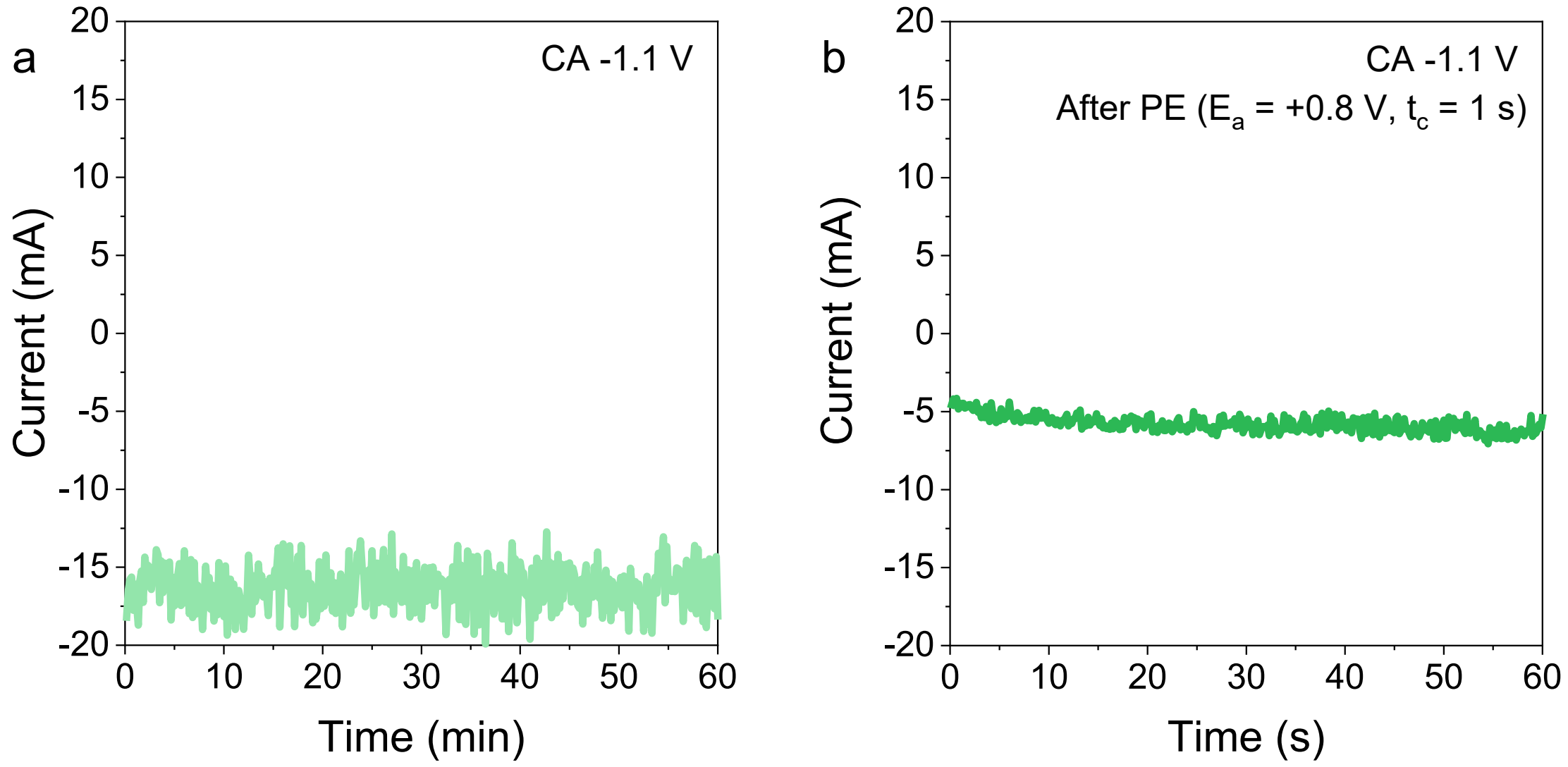

Figure S1. Current profile over time of the oxide-derived Cu catalyst during **(a)** the chronoamperometry (CA) at -1.1 V vs. RHE (no pulse) and **(b)** during the CA -1.1 V vs. RHE after pulsed electrolysis (PE) with E_a = +0.8 V, E_c = -1.1 V and t_a = t_c = 1 s for 1 h. The loss of the electrochemical surface area (ECSA) during PE explains the decrease in the current.

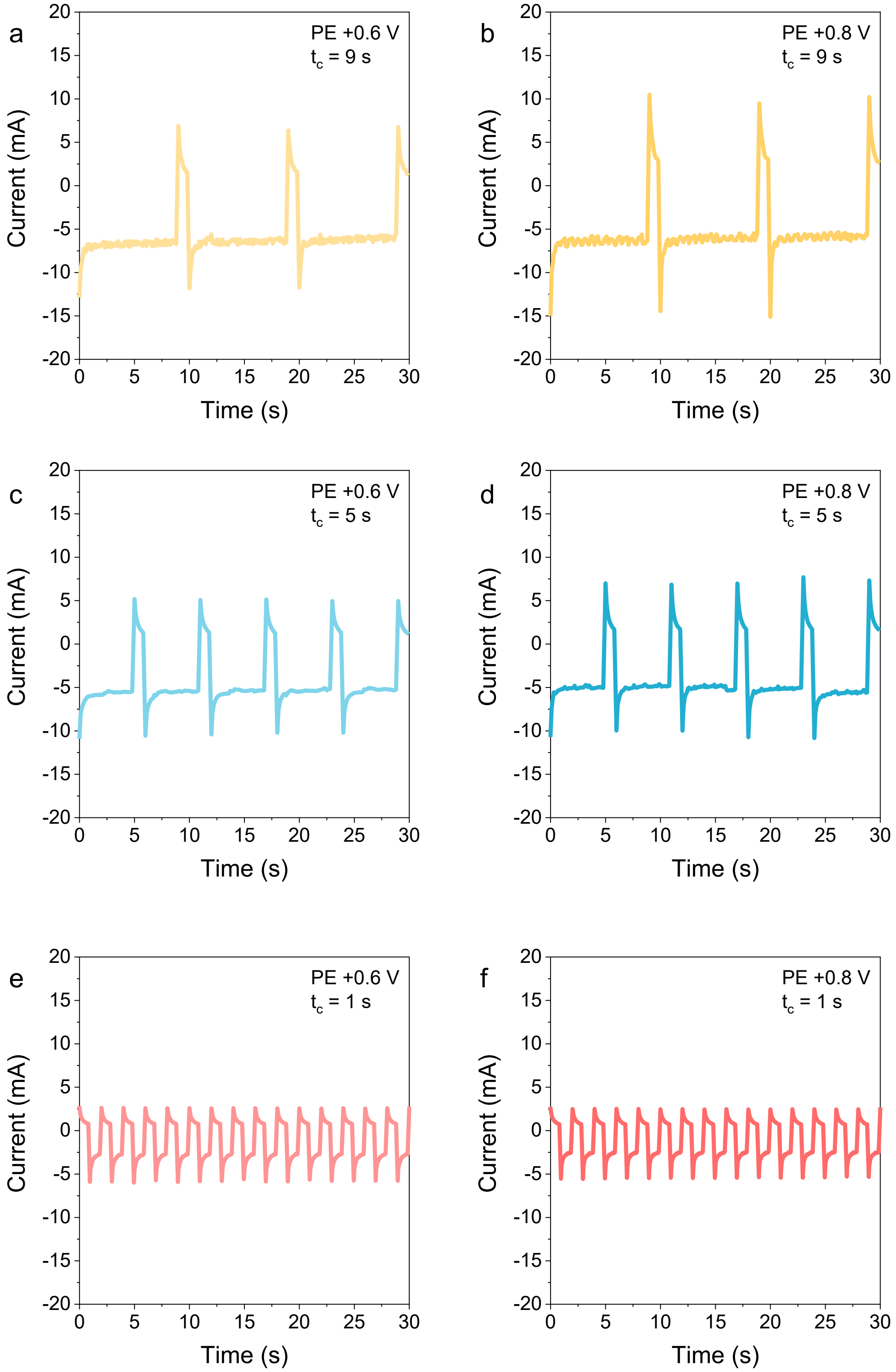

Figure S2. Current profile during the final 30 s of the PE measurements with **(a)** E_a = +0.6 V and t_c = 9 s, **(b)** E_a = +0.8 V and t_c = 9 s, **(c)** E_a = +0.6 V and t_c = 5s, **(d)** E_a = +0.8 V and t_c = 5 s, **(e)** E_a = +0.6 V and t_c = 1 s and **(f)** E_a = +0.8 V and t_c = 1 s. The cathodic pulse potential (E_c) and anodic pulse duration (t_a) were kept at -1.1 V and 1 s, respectively.

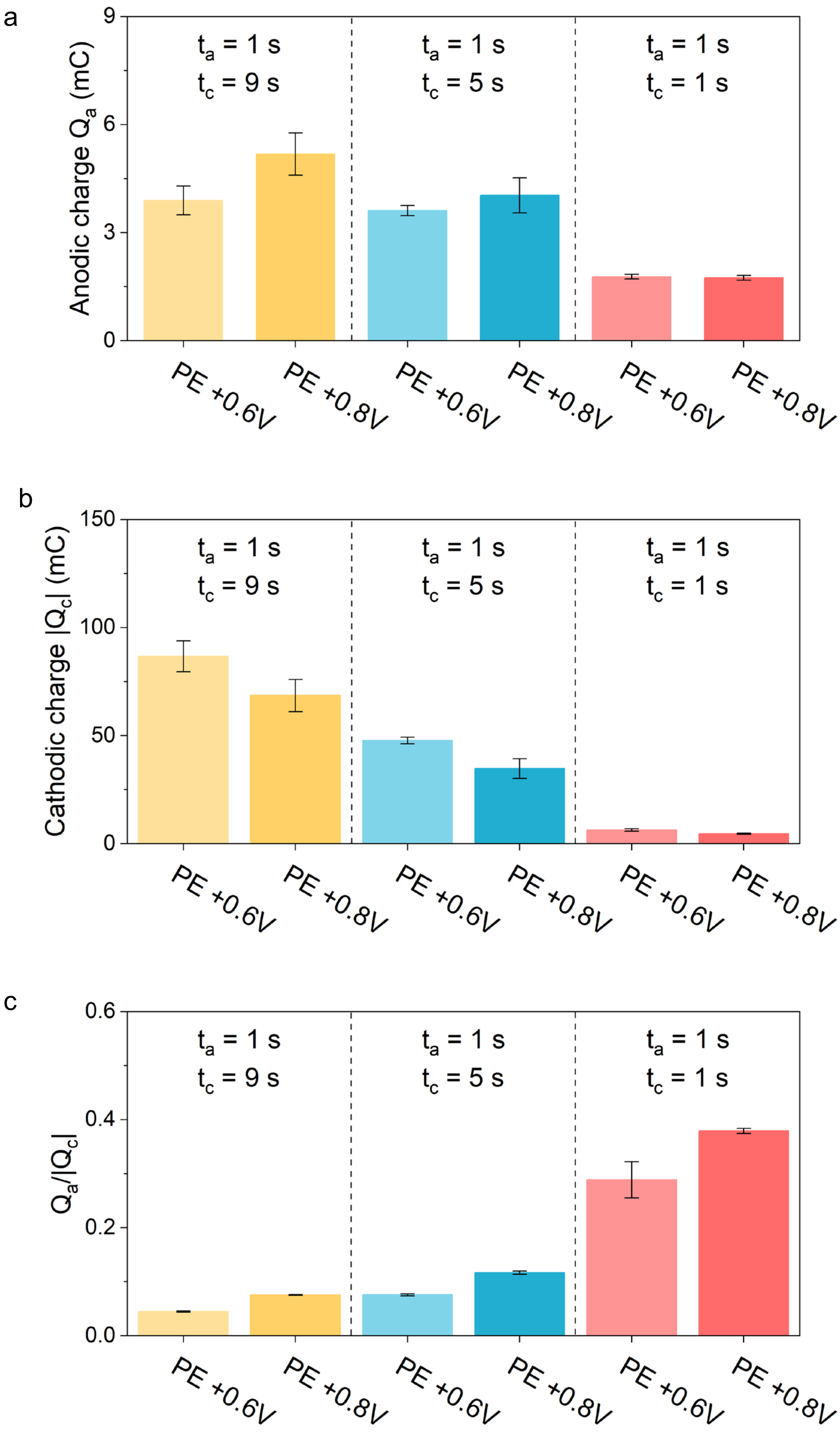

Figure S3. Average of the **(a)** anodic charge and **(b)** cathodic charge per cycle. **(c)** Ratio of the anodic charge over the cathodic charge per cycle.

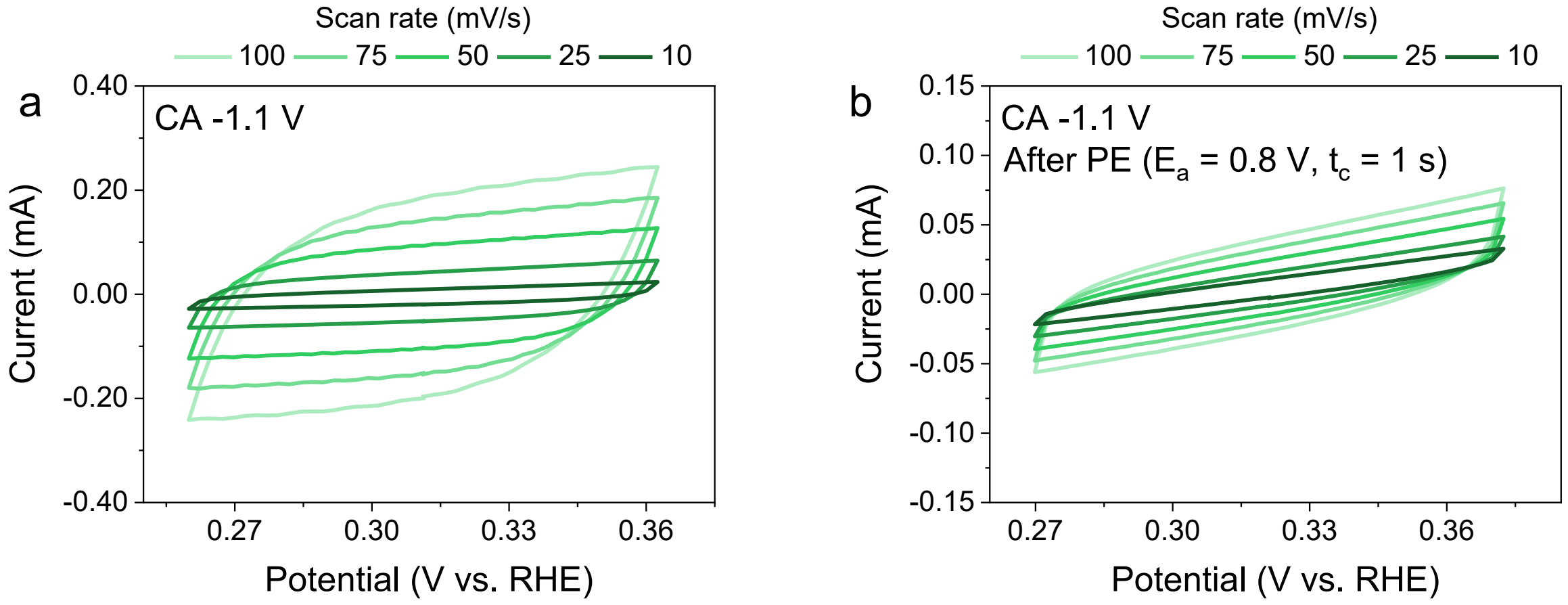

Figure S4. Examples of the ECSA measurements after **(a)** CA at -1.1 V and **(b)** pretreatment with PE (E_a = +0.8 V, E_c = -1.1 V and $t_a = t_c$ = 1 s) followed by CA at -1.1 V. A value of 40 μF cm^{-2} was used as a reference value and was obtained from literature.[1,2]

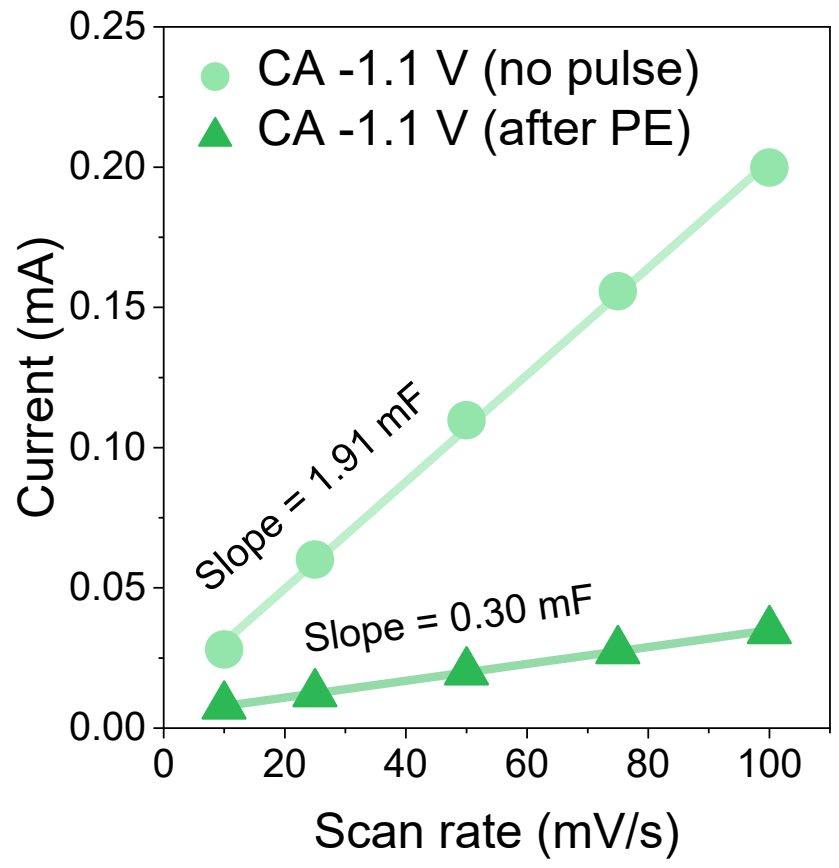

Figure S5. Slopes of the current vs. scan rate used for ECSA determination after CA at -1.1 V with and without PE pretreatment.

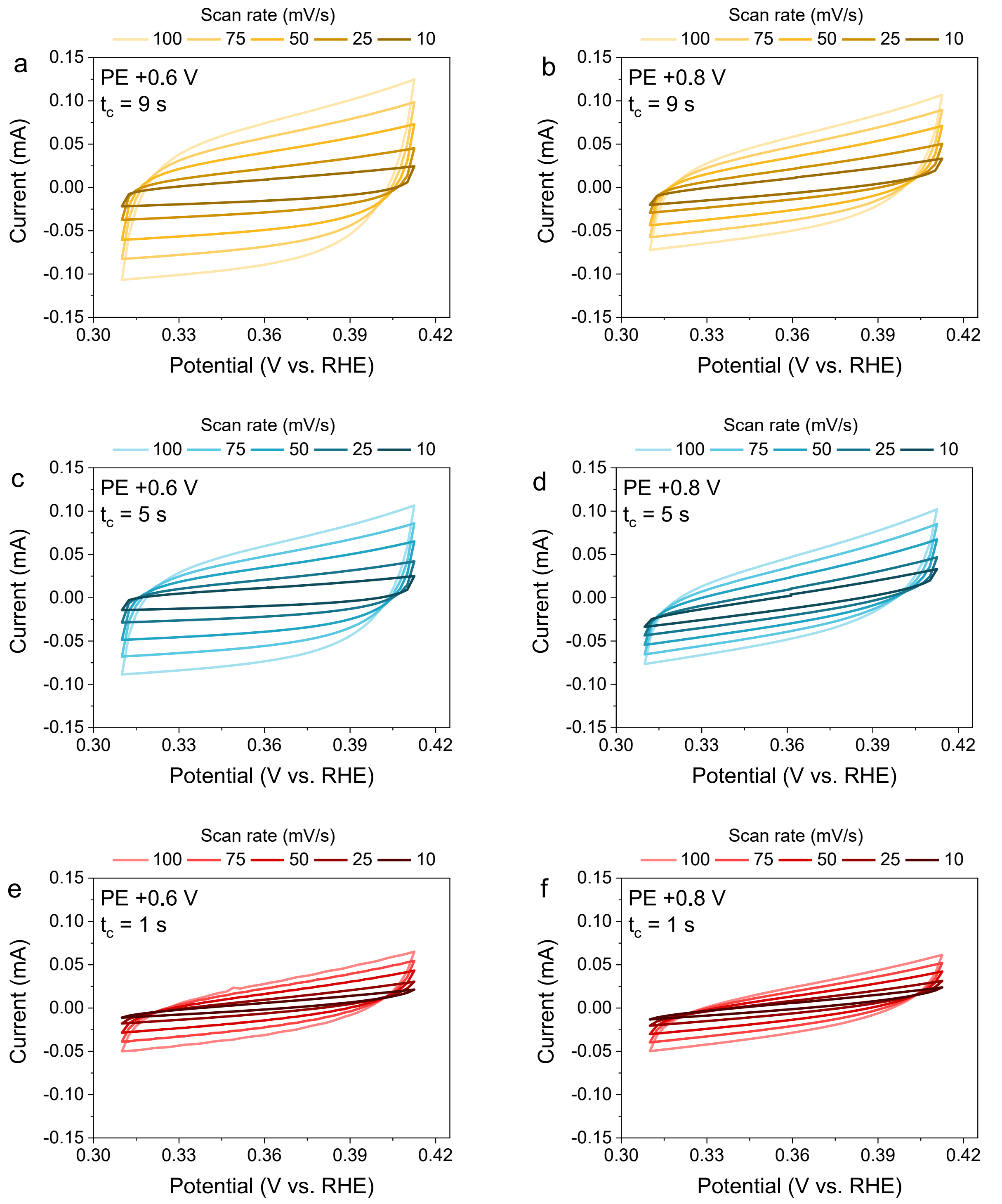

Figure S6. Examples of the ECSA measurements after PE with **(a)** E_a = +0.6 V and t_c = 9 s, **(b)** E_a = +0.8 V and t_c = 9 s, **(c)** E_a = +0.6 V and t_c = 5s, **(d)** E_a = +0.8 V and t_c = 5 s, **(e)** E_a = +0.6 V and t_c = 1 s and **(f)** E_a = +0.8 V and t_c = 1 s. The cathodic pulse potential (E_c) and anodic pulse duration (t_a) were kept at -1.1 V and 1 s, respectively.

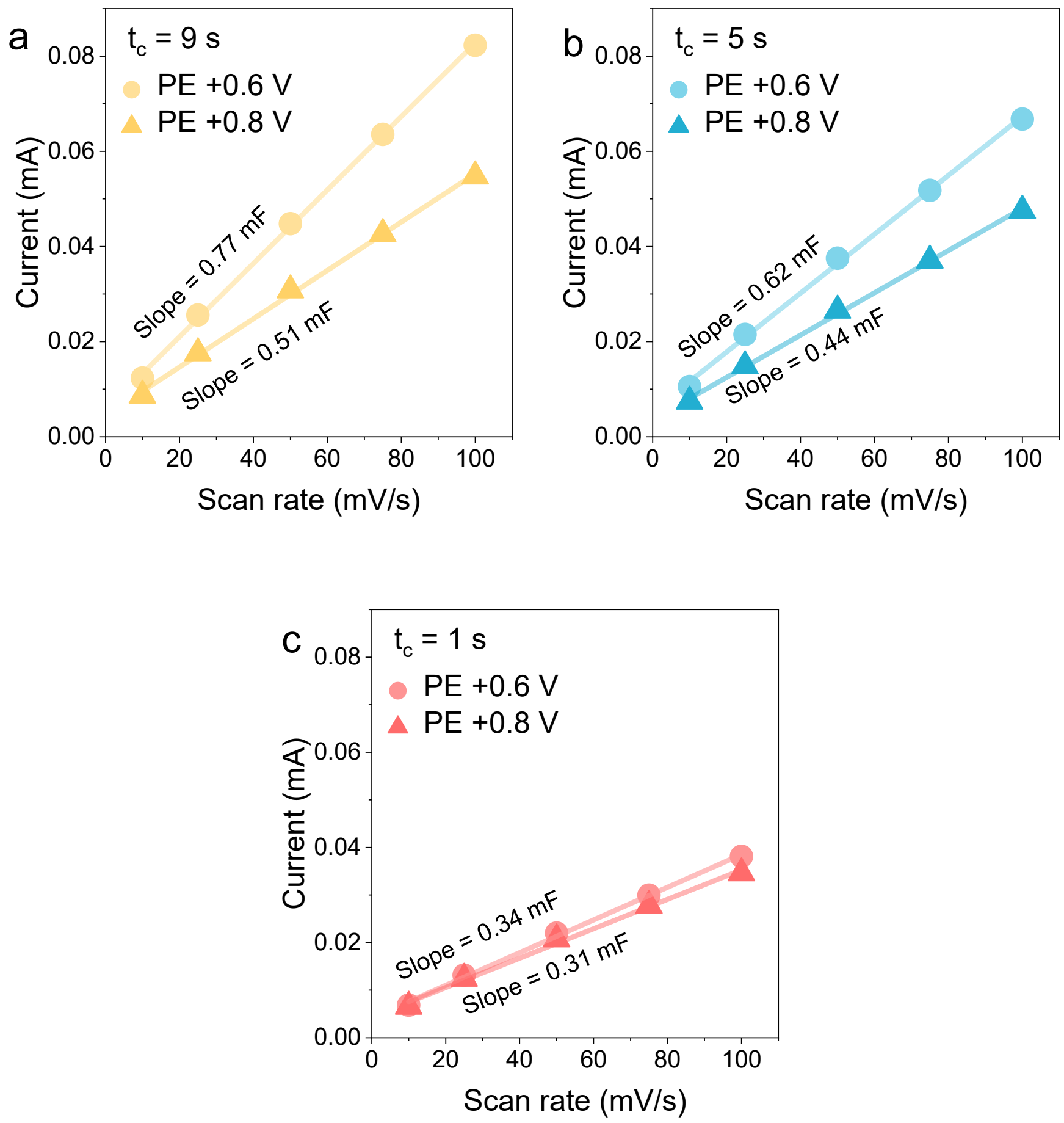

Figure S7. Slopes of the current vs. scan rate used for ECSA determination after PE +0.6 V and PE +0.8 V with t_c is **(a)** 9 s, **(b)** 5 s and **(c)** 1 s. The cathodic pulse potential (E_c) and anodic pulse duration (t_a) were kept at -1.1 V and 1 s, respectively.

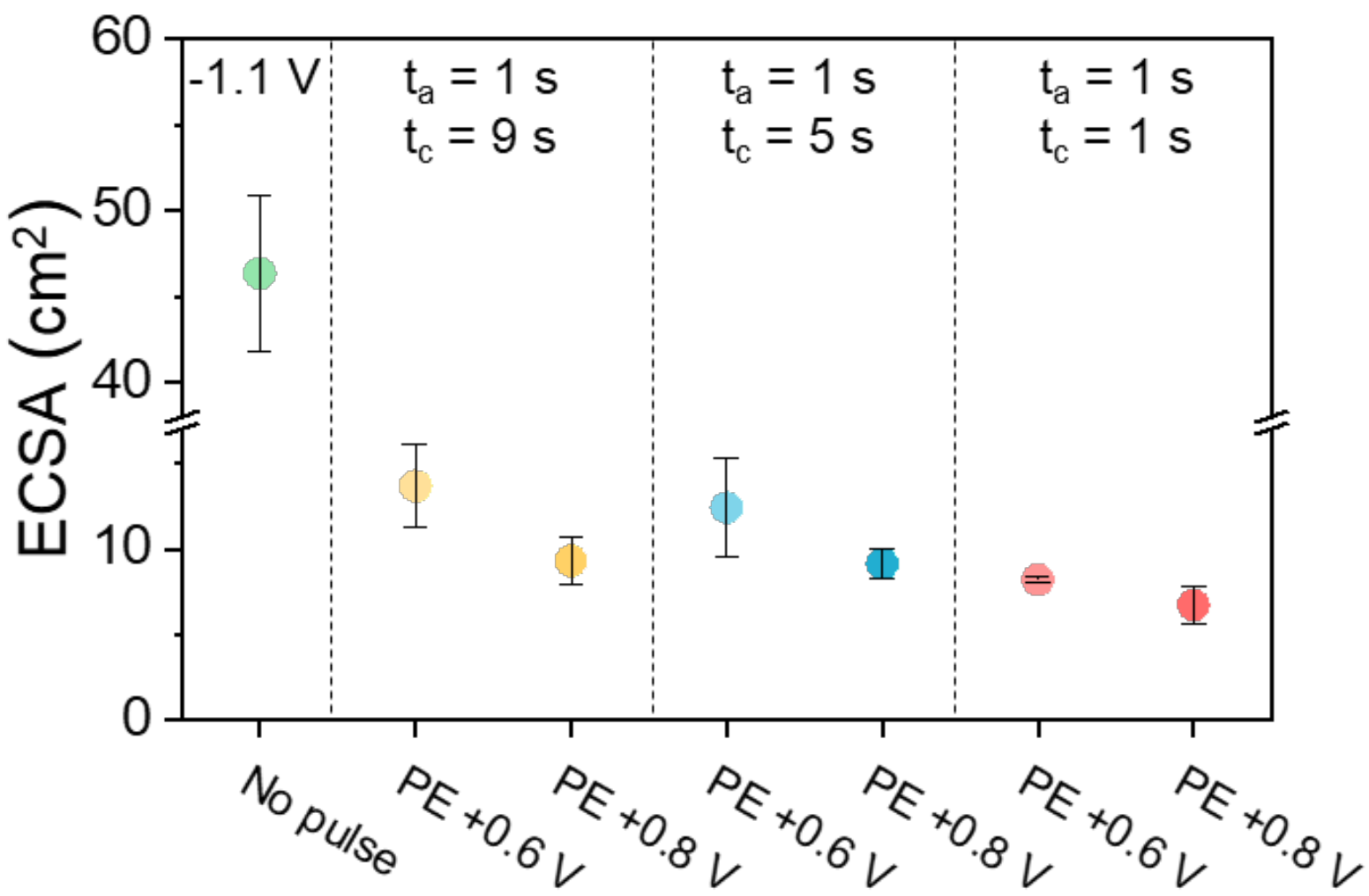

Figure S8. Values for the ECSA after (pulsed) CO_2RR measurements.

Table S1. Values for the double layer capacitance for each condition.

Sample	C_{dl} (mF/cm^2)
**CA -1.1 V**	1.86 ± 0.18
**PE +0.6 V t_c = 9 s**	0.56 ± 0.16
**PE +0.8 V t_c = 9 s**	0.37 ± 0.10
**PE +0.6 V t_c = 5 s**	0.50 ± 0.20
**PE +0.8 V t_c = 5 s**	0.37 ± 0.06
**PE +0.6 V t_c = 1 s**	0.33 ± 0.01
**PE +0.8 V t_c = 1 s**	0.28 ± 0.06

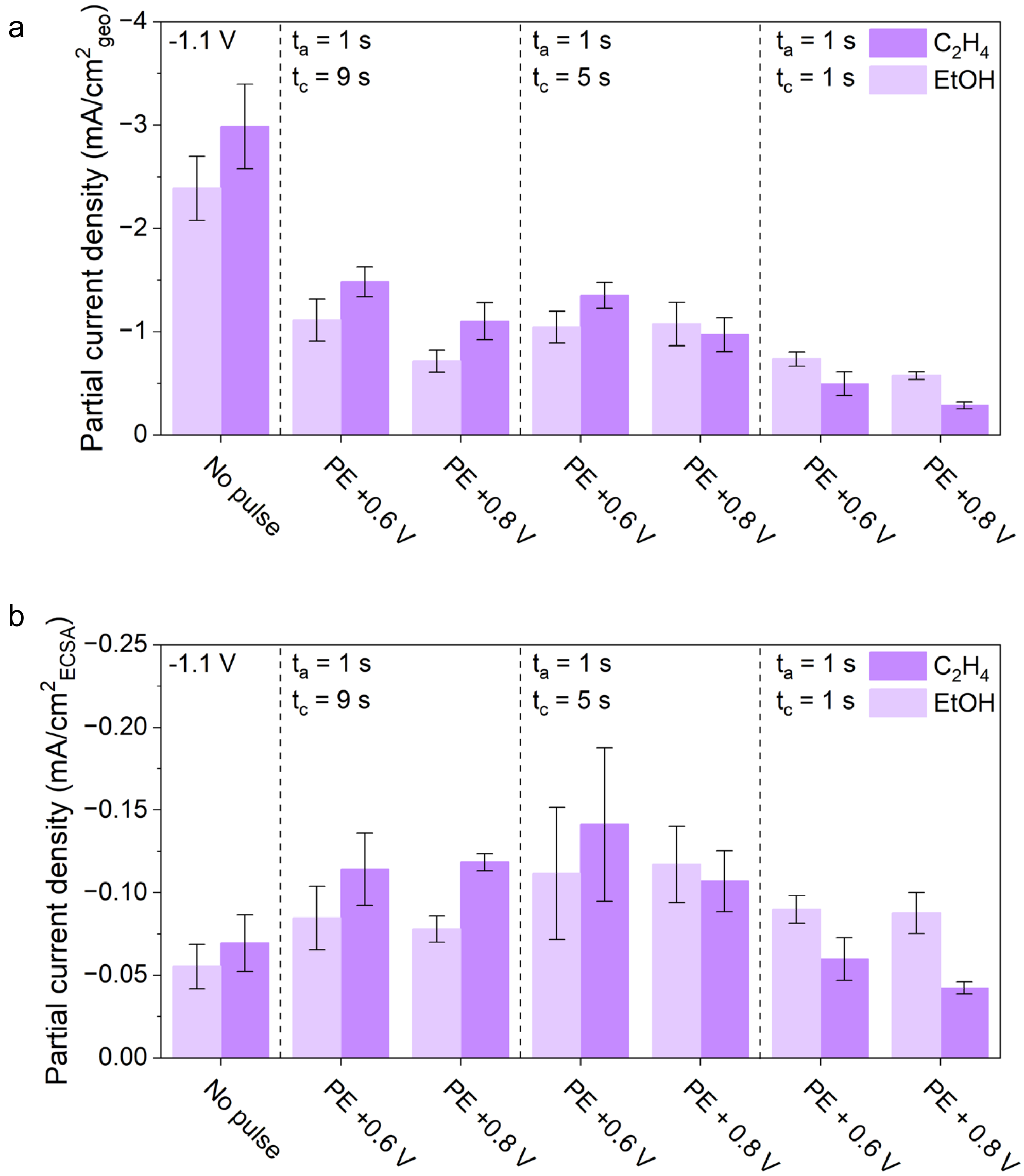

Figure S9. Partial current densities for ethylene and ethanol normalized by **(a)** geometric area and **(b)** electrochemical surface area.

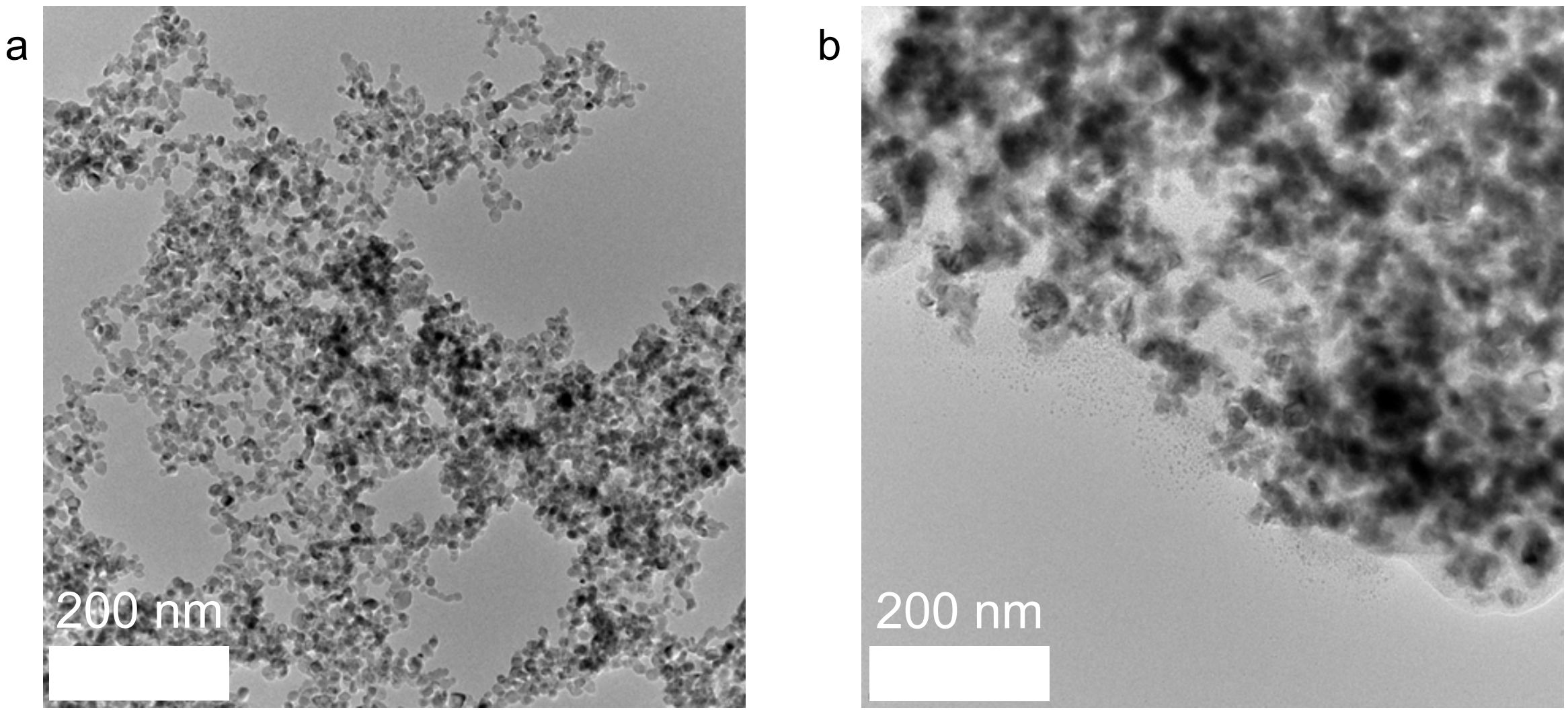

Figure S10. TEM image of the **(a)** as-prepared CuO catalyst and **(b)** after the CA at -1.1 V vs. RHE (no pulse) for 1 h.

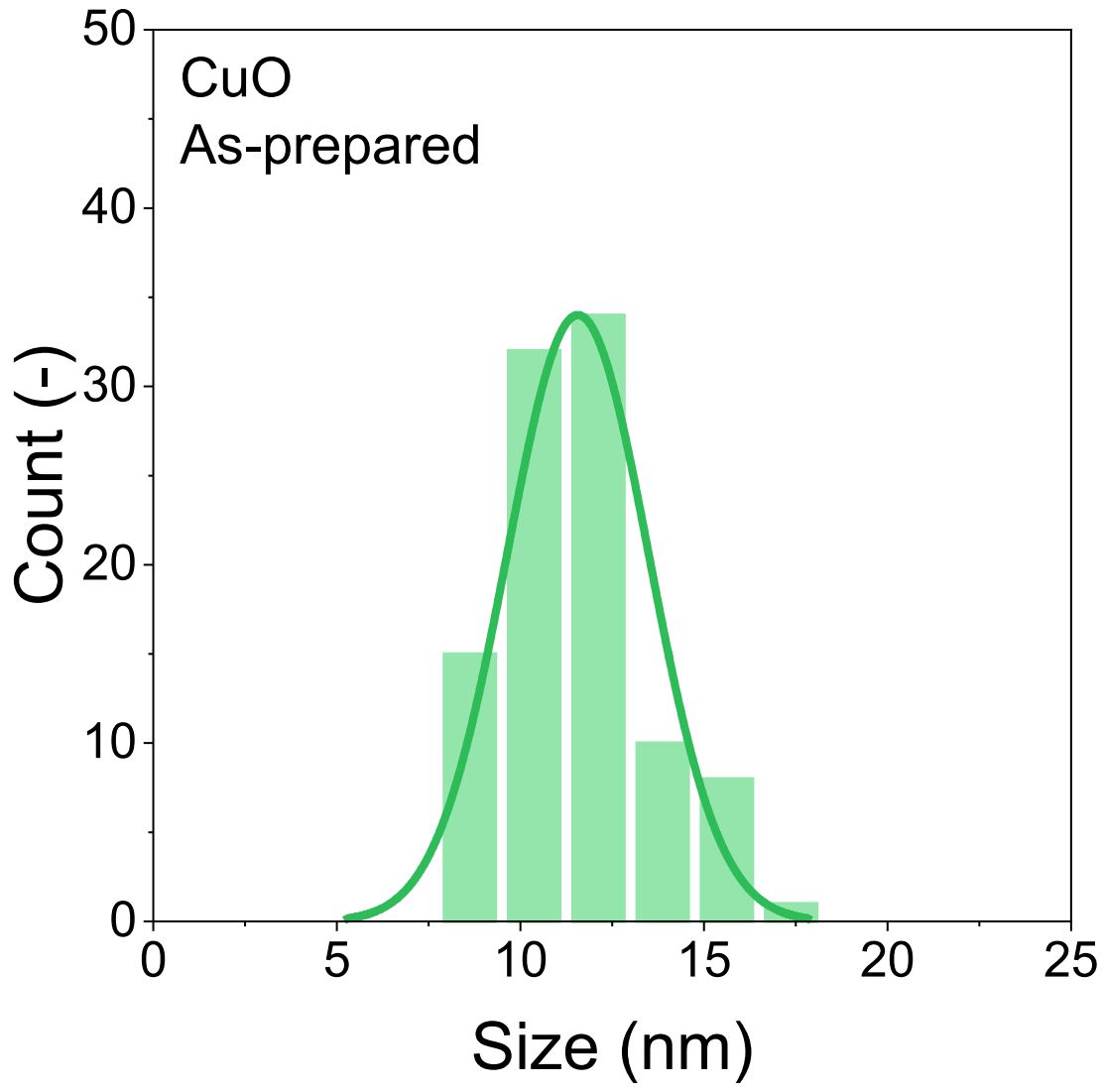

Figure S11. Particle size distribution of the as-prepared CuO catalyst.

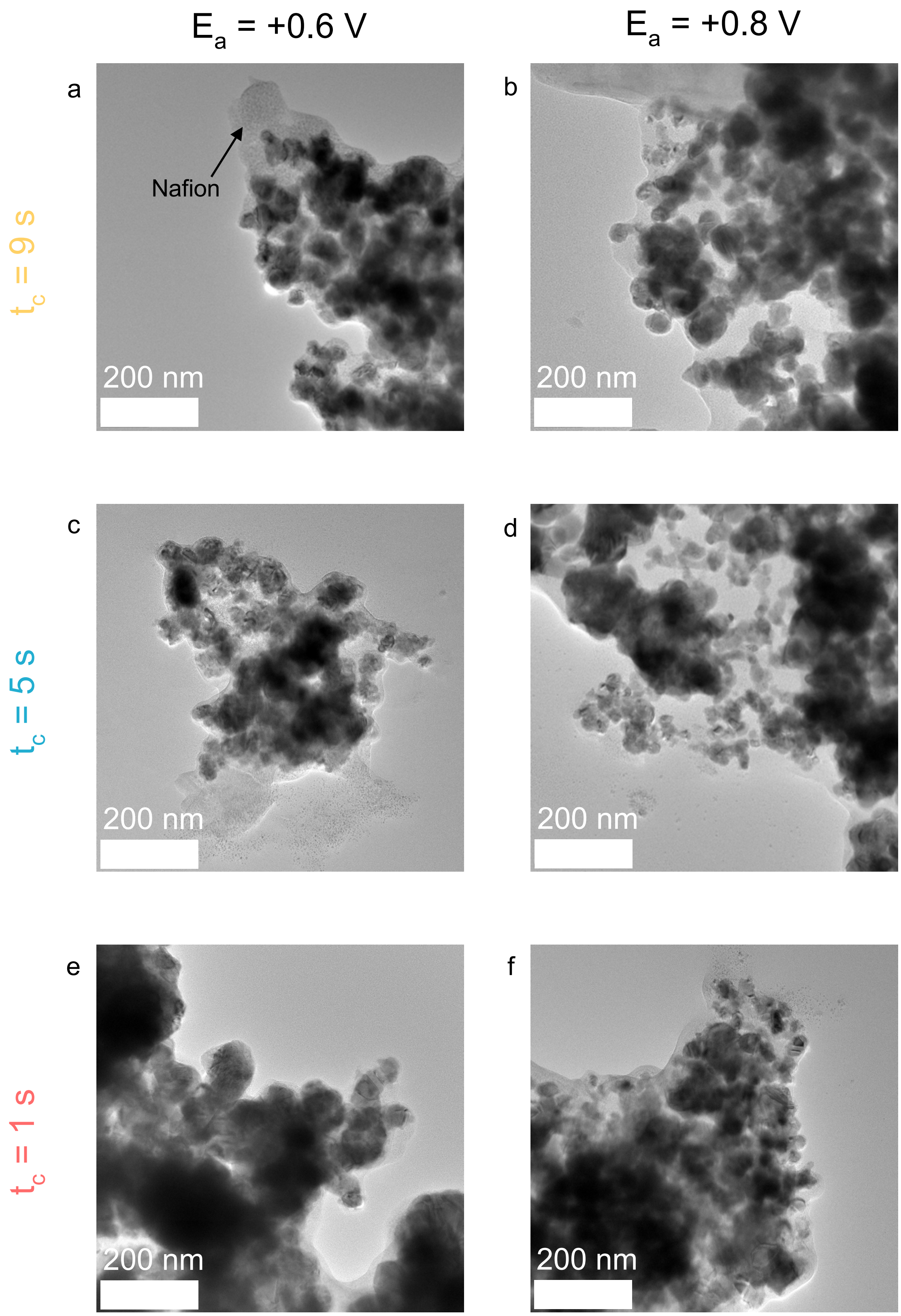

Figure S12. TEM images after PE with **(a)** E_a = +0.6 V and t_c = 9 s, **(b)** E_a = +0.8 V and t_c = 9 s, **(c)** E_a = +0.6 V and t_c = 5s, **(d)** E_a = +0.8 V and t_c = 5 s, **(e)** E_a = +0.6 V and t_c = 1 s and **(f)** E_a = +0.8 V and t_c = 1 s. The cathodic pulse potential (E_c) and anodic pulse duration (t_a) were kept at -1.1 V and 1 s, respectively.

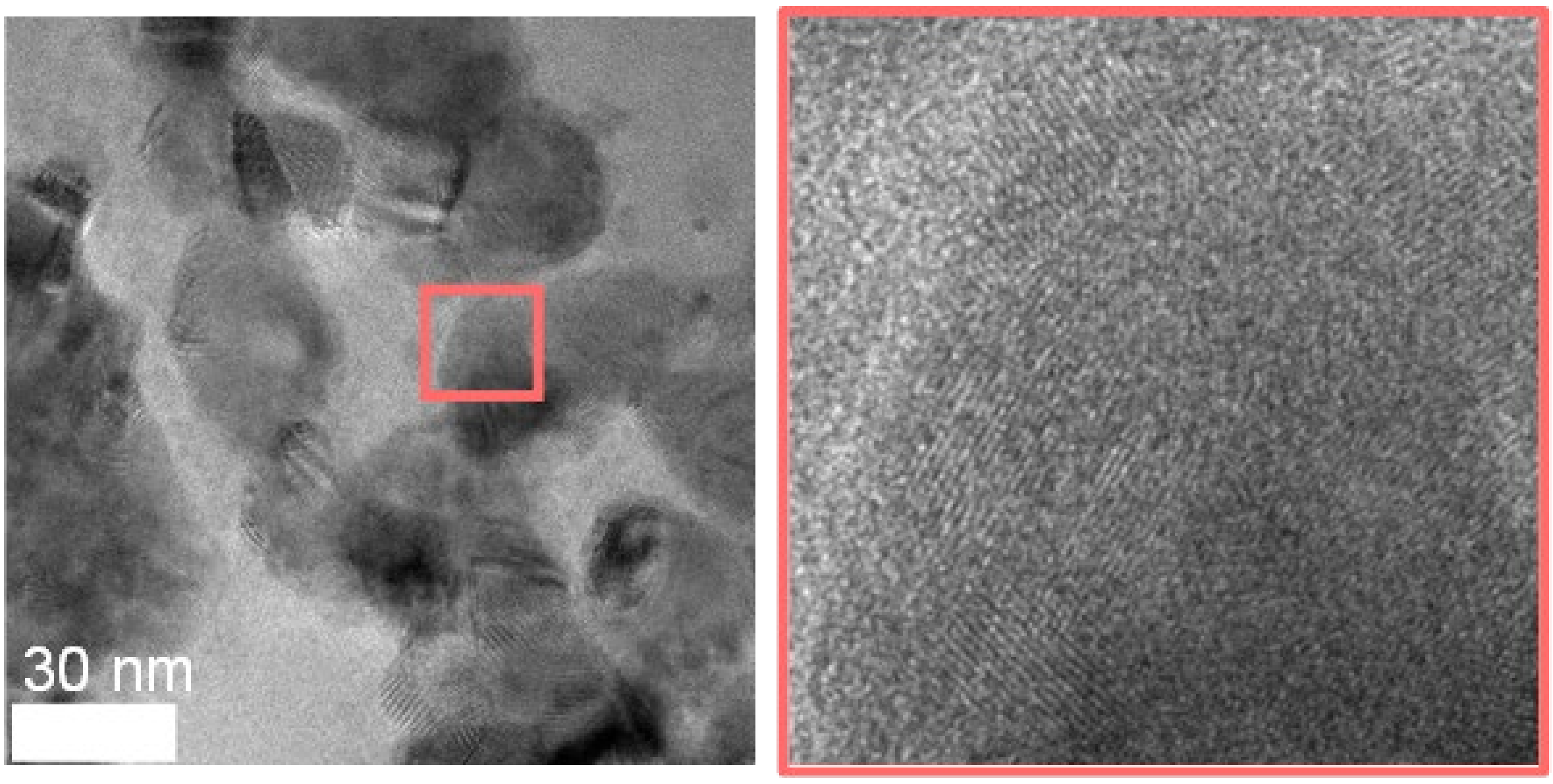

Figure S13. TEM image of PE +0.8 V with $t_a = t_c = 1$ s and $E_c = -1.1$ V. The area indicated by the red box (left) is shown in more detail in the right image and reveals the formation of grain boundaries.

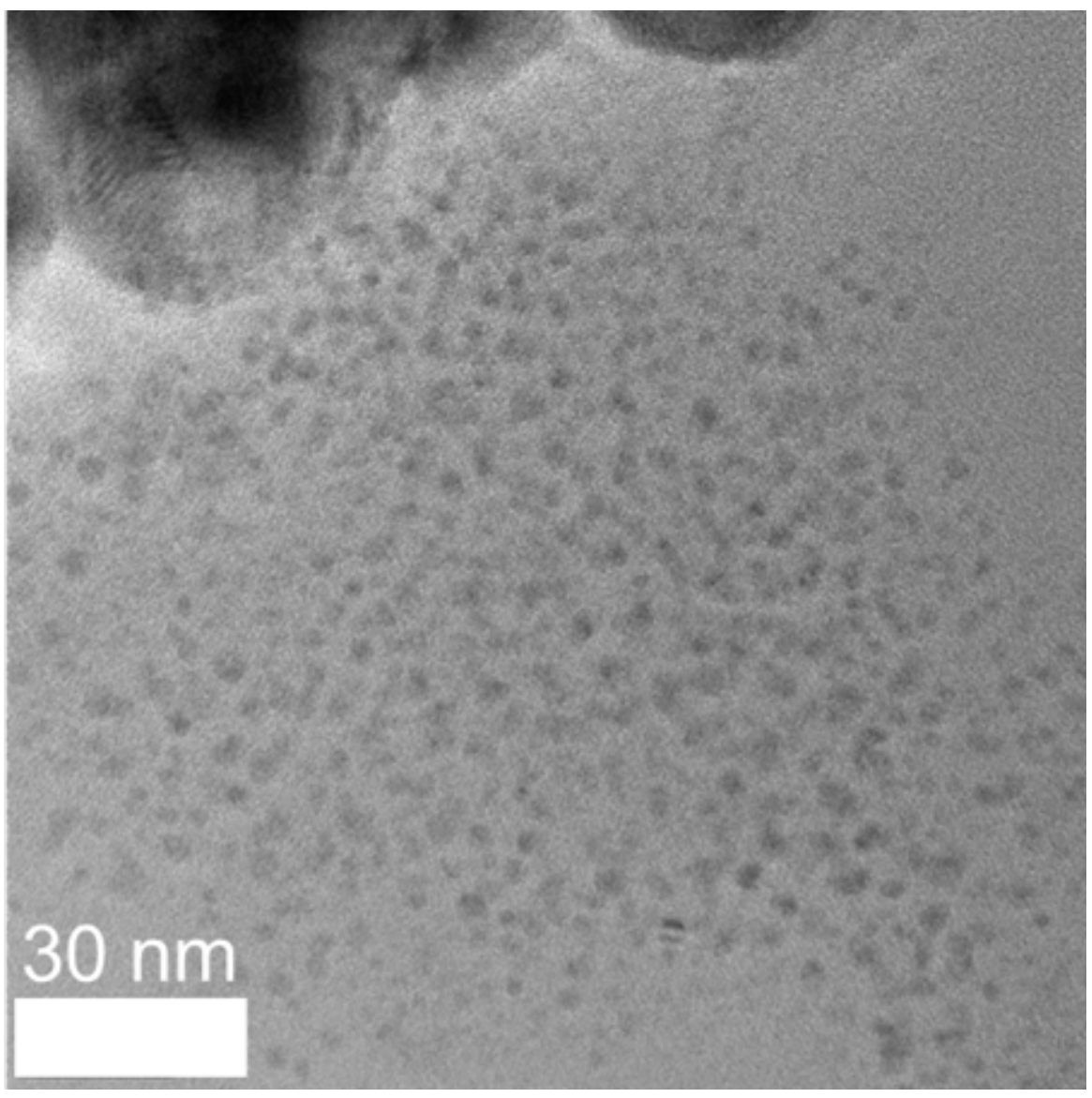

Figure S14. TEM image of PE +0.8 V with $t_a = t_c = 1$ s and $E_c = -1.1$ V. Besides sintering, small electrodeposits were observed.

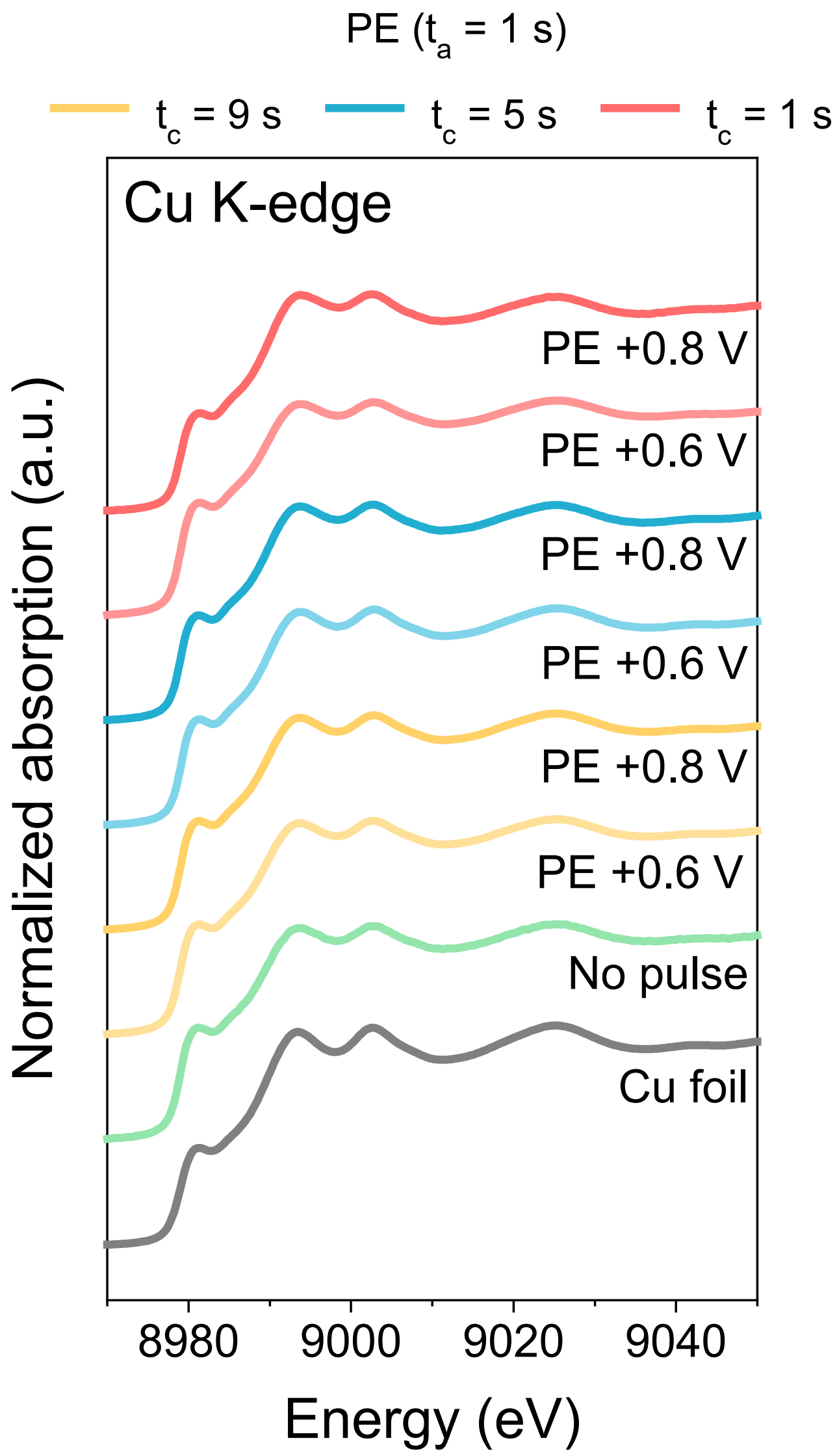

Figure S15. Final recorded XANES spectra during the (pulsed) CO_2RR for 1 h. Cu foil reference (grey) is shown for comparison.

Table S2. EXAFS fitting parameters. The data is measured in fluorescence mode. An S_0^2 value of 0.9 was calculated from fitting of the Cu foil reference and was used for the fitting of FT-EXAFS spectra. The calculations were carried out using the software Artemis.

Sample	Path	R (Å)	CN (-)	σ^2 ($Å^2$)	ΔE_0 (eV)	R-factor (%)
**CuO (ref.)**	Cu-O (oxide)	1.952(4)	4.4(4)	0.0049(12)	0.5(13)	0.68
**Cu foil (ref.)**	Cu-Cu (metal)	2.544(3)	12.0(5)	0.0087(3)	-0.1(4)	0.08
**CuO as-prepared**	Cu-O (oxide)	1.950(3)	2.9(3)	0.0025(11)	1.4(12)	0.36
**CA -1.1 V**	Cu-Cu (metal)	2.540(3)	8.5(4)	0.0080(4)	0.2(5)	0.08
**PE +0.6 V t_c = 9 s**	Cu-Cu (metal)	2.541(3)	8.7(4)	0.0080(4)	0.2(4)	0.09
**PE +0.8 V t_c = 9 s**	Cu-Cu (metal)	2.541(3)	9.1(4)	0.0080(4)	0.3(5)	0.07
**PE +0.6 V t_c = 5 s**	Cu-Cu (metal)	2.540(3)	9.4(5)	0.0078(4)	0.3(5)	0.11
**PE +0.8 V t_c = 5 s**	Cu-Cu (metal)	2.541(3)	9.0(4)	0.0079(4)	0.2(5)	0.08
	Cu-O (oxide)	1.88(7)	0.13(11)	0.003(1)	0.2(5)	0.08
**PE +0.6 V t_c = 1 s**	Cu-Cu (metal)	2.540(3)	8.3(4)	0.0080(4)	0.3(5)	0.09
	Cu-O (oxide)	1.9(7)	0.01(10)	0.003(3)	0.3(5)	0.09
**PE +0.8 V t_c = 1 s**	Cu-Cu (metal)	2.540(3)	9.0(4)	0.0079(4)	0.1(5)	0.09
	Cu-O (oxide)	1.84(3)	0.29(11)	0.003(1)	0.1(5)	0.09

References

(1) McCrory, C. C. L.; Jung, S.; Peters, J. C.; Jaramillo, T. F. Benchmarking heterogeneous electrocatalysts for the oxygen evolution reaction. *J. Am. Chem. Soc.* **2013**, 135, 16977–16987.

(2) Hegde, C.; Sun, X.; Dinh, K.N.; Huang, A.; Ren, H.; Li, B.; Dangol, R.; Liu, C.; Wang, Z.; Yan, Q.; Li, H. Cu- And Fe-Codoped Ni Porous Networks as an Active Electrocatalyst for Hydrogen Evolution in Alkaline Medium. *ACS Appl. Mater. Interfaces* **2020**, 12, 2380–2389.
